# Targeted delivery of berberine *via* ROS-sensitive polymersomes enhances its hepatoprotective activity in CCl_4_-intoxicated mice

**DOI:** 10.1039/d5na00706b

**Published:** 2025-12-03

**Authors:** Iva Suman, Damir Klepac, Martina Vragović, Hrvoje Križan, Eliézer Jäger, Alessandro Jäger, Ewa Pavlova, Martin Hrubý, Robert Domitrović

**Affiliations:** a Department of Medical Chemistry, Biochemistry and Clinical Chemistry, Faculty of Medicine, University of Rijeka Braće Branchetta 20 51000 Rijeka Croatia robert.domitrovic@medri.uniri.hr; b Centre for Micro- and Nanosciences and Technologies, University of Rijeka Radmile Matejčić 2 51000 Rijeka Croatia; c Institute of Macromolecular Chemistry v.v.i., Academy of Sciences of the Czech Republic Heyrovsky Sq. 2 162 06 Prague 6 Czech Republic

## Abstract

Carbon tetrachloride (CCl_4_) metabolism results in the production of highly reactive free radicals and consequent liver tissue damage, making CCl_4_-induced liver injury an ideal model for studying drug delivery systems that respond to reactive oxygen species (ROS). Previously, we demonstrated the hepatoprotective activity of isoquinoline alkaloid berberine (BER) against CCl_4_-induced hepatotoxicity in mice. In this study, we aimed to investigate the targeted delivery of BER to ROS-rich injury site. For this purpose, ROS-responsive polymersomes (PS), built as amphiphilic block copolymers bearing a boronic ester-based ROS sensor connected to the hydrophobic polymer backbone with embedded BER, were synthesized in our laboratory. PS exhibited a suitable particle size of 117.8 nm, zeta potential of −12.5 mV, and good physical stability. Mice were administered berberine (BER) and polymersome nanoencapsulated berberine (BER-PS) 6 mg kg^−1^ intraperitoneally, 1 h before CCl_4_ (10% v/v in olive oil, 2 mL kg^−1^) and sacrificed 48 h later. Serum levels of alanine aminotransferase (ALT) and aspartate aminotransferase (AST) were markedly decreased and histopathological changes were significantly reduced by BER-PS compared to BER. The expression of oxidative stress markers (4-hydroxynonenal (4-HNE), hem oxygenase-1 (HO-1), 8-hydroxy-2′-deoxyguanosine (8-OHdG)), apoptosis (caspase-3, caspase-9, TUNEL), autophagy (microtubule-associated protein 1 light chain 3 beta (LC3B)-I/II, p62), and inflammation (tumor necrosis factor-alpha (TNF-α), nuclear factor kappa B (NF-κB)) was also more effectively ameliorated by BER-PS. Mechanistically, both BER and BER-PS decreased the expression of phosphorylated extracellular signal-regulated kinase (ERK)1/2 and phosphorylated AMP-activated protein kinase (AMPK). BER-PS also decreased nuclear factor-kappa B (NF-κB), tumor necrosis factor-alpha (TNF-α), phosphorylated c-Jun N-terminal kinase (JNK)1/2 and phosphorylated protein kinase B (Akt). These results suggest that BER-PS is more successful than BER in ameliorating ROS-mediated CCl_4_-induced hepatic injury, which could be related to the specifically targeted delivery of the drug to the site of injury under oxidative stress conditions.

## Abstract 2

Carbon tetrachloride (CCl_4_) metabolism results in the production of free radicals and consequent liver tissue damage, making CCl_4_-induced liver injury an ideal model for studying drug delivery systems that respond to reactive oxygen species (ROS).

## Graphical abstract

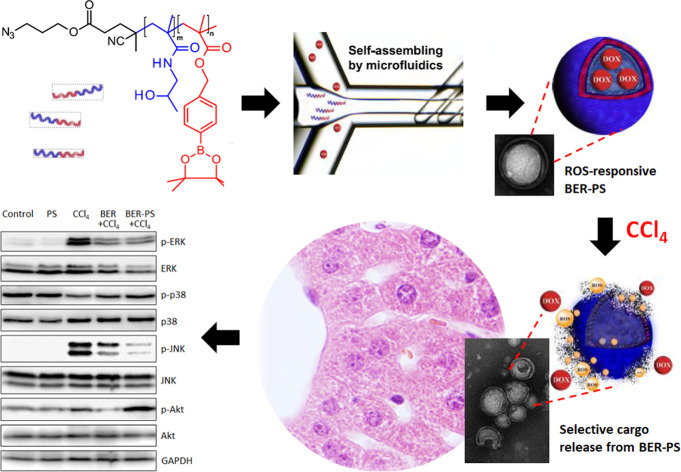

## 1. Introduction

The liver is the central organ in the metabolism of xenobiotics and the primary target for drugs and toxins.^1^ Carbon tetrachloride (CCl_4_) is a highly hepatotoxic industrial solvent, frequently used as a model of toxic liver injury. The toxicity of this compound is attributed to trichloromethyl radicals (˙CCl_3_) and trichloromethyl peroxyl radicals (˙CCl_3_O_2_), which are generated during its metabolism in the liver.^2^ These radicals can interact with cellular macromolecules, impairing crucial cellular processes.

Natural compounds could prevent the deleterious effects of reactive oxygen species (ROS) and free radicals, acting as antioxidants, but also by modulating cellular response to damage independently of their antioxidant behavior.^3^ Berberine (BER) is an isoquinoline alkaloid of the protoberberine type found in the root, rhizome, and stem bark of numerous plant species mainly from Berberidaceae family.^4^ Extensive research on berberine's pharmacologic properties revealed antioxidative, antimicrobial, anti-inflammatory, anticancer, and multi-organ-protective activities.^5^ Previously, we showed strong hepatoprotective activity of BER in CCl_4_-intoxicated mice.^6^ Despite the reported activities, BER limited solubility and consequently limited bioavailability restrict its clinical application.^7^

Cell delivery of biologicaly active molecules is critical for advancing biomedical research and therapeutic applications.^8^ Significant progress in nanomedicine has been made recently, including liposomes, micelles, dendrimers, carbon nanotubes, metal-based nanoparticles, well as polimersomes (PS), in order to enhance drug delivery to the cells.^9,10^ Nanoparticles can be also combined with other delivery systems, such as nanofibers.^11^

Liposomes serve as excellent drug delivery vehicles because of their ability to readily degrade in the body. They can encapsulate hydrophilic substances in their internal aqueous compartments and lipophilic substances in their surrounding lipid membranes.^12,13^ Carbon nanotubes are usually chemically modified to increase their water solubility, and they are used to deliver therapeutics such as proteins, peptides, nucleic acids, and other active molecules.^14^ Dendrimers can encapsulate many therapeutic agents owing to their branched structures. Similar to polymersomes, dendrimers can be modified to acquire stimuli-responsive properties and release drugs in response to specified triggers, such as pH and redox environments.^15^ Among various delivery systems, polymersomes are considered attractive nanomedicine platforms because they can encapsulate both hydrophobic and hydrophilic drugs with a higher loading capacity than liposomes.^16^

Various nanomedicine delivery systems have also been explored for the treatment of liver injury. Extracellular vesicles that have been modified to express signal-regulatory protein alpha have shown significant therapeutic potential in addressing acute liver failure by specifically targeting damaged cells and facilitating tissue repair.^17^ The liposomal nanodrug containing itaconate exhibited a preference for liver accumulation and effectively mitigated lipopolysaccharide/d-galactosamine-induced liver histopathological damage by lowering oxidative stress levels.^18^ Bovine serum albumin nanoparticles loaded with silibinin demonstrated excellent hepatoprotective and antioxidant properties against acute liver injury both *in vivo* and *in vitro*.^19^ Poly(acrylic) acid-coated Mn_3_O_4_ nanoparticles have been found to alleviate acute liver injury by inhibiting ferroptosis through multifaceted mechanisms.^20^ Ultrasmall ruthenium nanoparticles have been demonstrated to sustainably alleviate oxidative stress and promote the upregulation of regulatory T cells in advanced stages of acetaminophen-induced liver injury.^21^

The enhanced stability and higher loading capacity of PS represent advantages compared to other systems, which has led to the development of novel PS in recent years.^9^ Their properties can be fine-tuned by synthesizing block copolymers with different molecular weights. Moreover, the possibility to easily functionalize PS and make them susceptible to microenvironments, such as redox potential, represents the next step in the development of smart nanomedicines. Possible limitations of PS compared to other delivery systems are reduced permeability and fluidity compared to liposomes, reproducible production, potential low biocompatibility, and difficulty in loading large biomolecules like therapeutic proteins.^22^

In this study, we investigated the hepatoprotective activity of novel berberine-loaded ROS-sensitive polymersome nanoparticles (BER-PS) as an advanced drug delivery approach compared to free BER. We evaluated the advantages of the current drug delivery system by studying histopathological changes, oxidative stress, apoptosis, autophagy, and the molecular mechanisms of their protective activity in a model of free radical-mediated liver injury.

## 2. Experimental

### 2.1. Chemicals

Berberine was purchased from Polyphenols Laboratories AS (Sandnes, Norway). Carbon tetrachloride (CCl_4_), olive oil, and sodium dodecyl sulfate (SDS) were purchased from Sigma Chemical Co. (St. Louis, MO, USA). Diagnostic kits for alanine aminotransferase (ALT), and aspartate aminotransferase (AST) were obtained from DiaSys Diagnostic Systems (Holzheim, Germany). Primary antibodies for HRP-conjugated anti-mouse IgG were obtained from Sigma-Aldrich Chemie GmbH (Steinheim, Germany) and HRP-conjugated anti-rabbit IgG from Santa Cruz Biotechnology (Santa Cruz, CA, USA). The ROS-responsive block copolymer named poly[*N*-(2-hydroxypropyl)methacrylamide]-*b*-poly[4-(4,4,5,5-*tetra*-methyl-1,3,2-dioxaborolan-2-yl)benzyl methacrylate] (PHPMA_37_-*b*-PbAPE_42_, herein named ROS-responsive block copolymer) was synthesized as previously reported.^9^

### 2.2. Manufacture of the BER-loaded polymersomes (BER-PS)

BER-loaded PS were produced using a microfluidic device setup from Dolomite (Royston, United Kingdom) equipped with a glass Micromixer chip with 12 mixing stages micro-channels of 50 µm × 125 µm (depth × width). The ROS-responsive block copolymer was dissolved in THF/MeOH (80/20 v/v) at a concentration of 5.0 mg mL^−1^ as the organic phase (OP). The polymer solutions were pumped through the middle channel and water for injection (pH 7.4) through the side channels, as the water phase (WP) containing 1 mg of BER, using two independent Dolomite Mitos P-Pump (Royston, United Kingdom) controlled *via* computer software. The flow rates were 100 µL min^−1^ for the WP and 100 µL min^−1^ for the OP. The PS were collected and non-encapsulated BER and solvents were removed using Amicon® Ultra-4 Centrifugal filter units after several washing steps and concentrated to 1 mL.

### 2.3. Characterization of the BER-loaded polymersomes

#### 2.3.1. Dynamic light scattering (DLS)

Particle size measurements were conducted using a Zetasizer Nano ZS, Model ZEN3600 (Malvern Instruments, UK) equipped with a 633-nm He–Ne laser and operating at an angle of 173°. Dispersion Technology Software version 6.01 (Malvern) was used to collect and analyze the data. 1 mL of the PS (0.2 mg) was measured in single-use polystyrene half-micro cuvettes (Fisher Emergo, Landsmeer, The Netherlands) with a pathlength of 10 mm. The measurements were made at a position of 4.65 mm from the cuvette wall with an automatic attenuator at controlled temperature of 25 °C and 37 °C. For each sample, one run of 45 s was performed, with 10 repetitions for all PS. The Z-average diameter and polydispersity index (PDI) were obtained from the autocorrelation function using the “general purpose mode”. The error bars displayed on the DLS tables were obtained using the standard deviation (SD) of 10 measurements of the same sample.

#### 2.3.2. Electrophoretic light scattering (ELS)

The values of zeta potential (*ζ*) of the produced polymer colloids were collected using a Zetasizer Nano ZS ZEN3600 instrument (Malvern Instruments, UK) which measures the electrophoretic mobility (UE) and converts in to *ζ*-potential (mV) using Henry's equation:1
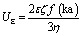
where *ɛ* is the dielectric constant of the medium, *η* its viscosity, and *f*(ka) is the Henry's function calculated using the Smoluchowski approximation (*f*(ka) = 1.5).

#### 2.3.3. Cryo-transmission electron microscopy (cryo-TEM)

Cryo-TEM images were acquired using a FEI Tecnai G2 Spirit TWIN microscope in bright-field imaging mode at an accelerating voltage of 120 kV. Four-µL of the samples were loaded into electron microscopy grids covered with a holey or lacey carbon supporting film (electron microscopy science), which were hydrophilized just before the experiment *via* glow discharge (Expanded Plasma Cleaner, Harrick Plasma, USA). The excess samples were removed by blotting (Whatman no. 1 filter paper) and the grids were plunged into liquid ethane held at −182 °C. The vitrified samples were then immediately transferred to the microscope and observed at −173 °C. Image analysis was performed using ImageJ software.

### 2.4. BER encapsulation and release behavior

The BER content loaded into the PS was determined by using HPLC (UV = 270 nm). The BER loading content (LC) and BER encapsulation efficiency (EE) were determined using the following equations:2
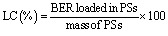
3
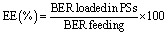


The BER release experiments were performed using the dialysis method according to previously published methodologies^23,24^ under two different environmental conditions: phosphate-buffered saline (PBS) (pH 7.4) only and in the presence of 1 mM H_2_O_2_. A pre-swollen cellulose dialysis membrane tube with a MWCO of 6–8 kDa (Pur-A-LyzerTM) was filled with 2.0 mL of BER-loaded PS at 0.5 mg mL^−1^. The membrane tube was then immersed in 3 L of different release media at 37 °C under stirring (350 rpm). At predetermined times, 500 µL of the BER-loaded PS was sampled from the inner compartment and the remaining BER amount in the samples was measured and determined by UV-vis spectroscopy as mentioned above. The sampled amount was then returned to the corresponding membrane tube.

### 2.5. *In vivo* study

Male Balb/C mice from our breeding colony, 2–3 months old, were randomly divided into 5 groups (control, PS, CCl_4_, CCl_4_ + BER, and CCl_4_ plus BER-PS) with 6 animals per group and maintained as previously described.^6^ BER (6 mg kg^−1^) dissolved in DMSO and diluted with saline (the final DMSO solution 5% v/v) was administered intraperitoneally (i.p.) 1 h before CCl_4_ dissolved in olive oil (i.p., 10% v/v, 2 mL kg^−1^). PS and BER-PS were diluted in distilled water and 5% DMSO was added. The control and PS-treated groups received 5% DMSO in saline. The CCl_4_ group received 5% DMSO and CCl_4_ 1 h later. The BER dose was selected based on our previous study.^6^ In the current study, which is an extension of the previous one, we used a lower dose of BER and an equal dose of nanoencapsulated BER to observe the hepatoprotective potential of BER-PS.

Free BER has a fast blood clearance, followed by rapid biotransformation in the liver.^25^ In contrast, BER-PS slowly releases free BER.^9^ Therefore, we treated mice for 48 h to enable the release of BER at the site of injury. The mice were then anesthetized with ketamine and euthanized by cervical dislocation. Blood was collected from the retro-orbital sinus and serum was separated to determine ALT and AST activities. Liver samples were collected for western blotting and immunohistochemistry analyses. Animal procedures were approved by the Ethics Committee of the Faculty of Medicine, University of Rijeka, and followed the European Council Directive (2010/63/EU).

### 2.6. Serum markers of liver damage

Serum levels of ALT, AST, and ALP, markers of liver injury, were measured by using a Bio-Tek EL808 Ultra Microplate Reader (BioTek Instruments, Winooski, VT, USA) according to the manufacturer's instructions.

### 2.7. Histopathology

Liver tissues were processed routinely using standard techniques, as described previously.^6^ Liver necrosis was evaluated in paraffin tissue sections stained with hematoxylin and eosin (HE) by measuring the percentage of liver with destroyed hepatocytes using ImageJ software (version 1.54 g, National Institutes of Health).

### 2.8. Immunohistochemistry

Immunohistochemical (IHC) studies were performed in paraffin tissue sections by using primary antibodies against tumor necrosis factor-alpha (TNF-α) (ab1793, Abcam, Cambridge, UK) and nuclear factor kappa B (NF-κB) p65 subunit (ab7970, Abcam, Cambridge, UK, 1 : 1000), employing DAKO EnVision+ System, Peroxidase/DAB kit according to the manufacturer's instructions (DAKO Corporation, Carpinteria, CA, USA), as described previously.^6^ Briefly, deparaffinized and rehydrated sections were exposed to high-temperature antigen retrieval in citrate buffer solution (0.01 M, pH 6.0) for 20 min. Endogenous peroxidase activity was blocked using 3% hydrogen peroxide in methanol for 30 min. The slides were washed with PBS (pH 7.2) and incubated with 5% bovine serum albumin (BSA) in PBS for 1 h. The slides were incubated with a primary antibody against NF-κB in 1% BSA in PBS overnight at 4 °C in a humidified chamber. After washing and incubation with HRP-labeled secondary antibody, the slides were incubated with 3, 3-diaminobenzidine (DAB) substrate and counterstained with hematoxylin. Immunostaining intensity was examined using light microscopy (Olympus BX51, Tokyo, Japan). IHC staining intensity score was calculated using IHC profiler plugin in ImageJ software.^26^

### 2.9. Immunofluorescence

Paraffin-embedded tissues were cut into 4-µm thick sections and processed as described for immunohistochemical analysis. The slides were incubated with 1% BSA in Tris-buffered saline for 1 h. The blocking serum was removed and the sections were incubated with primary antibody against 8-hydroxy-2′-deoxyguanosine (8-OHdG) (sc-66036, Santa Cruz, CA, USA, 1 : 200) in a humidified chamber at 4 °C overnight. The slides were then incubated with mouse-IgGκ BP-CFL 594 (sc-516178, Santa Cruz, CA, USA, 1 : 200), covered with mounting medium and examined under fluorescent microscope (Olympus BX51, Tokyo, Japan). Immunoflurescence staining intensity scores were calculated using the ImageJ software.

### 2.10. TUNEL assay

Terminal deoxynucleotidyl transferase dUTP nick end labeling (TUNEL) is a method for detecting DNA fragmentation, a hallmark of apoptotic cell death, by labeling the 3′- hydroxyl termini in the double-strand DNA breaks generated during apoptosis. The TUNEL assay was performed according to the manufacturer's instructions (Proteintech, Rosemont, IL, USA). Briefly, kidney sections (4 µm thick) were immersed in xylene and ethanol, and then rehydrated in a decreasing ethanol gradient and washed. Tissues were permeabilized in 10 mM sodium citrate for 20 min at 95 °C. Slides were blocked in 0.1 M Tris–HCl pH 7.5 with 3% bovine serum albumin and 20% fetal bovine serum, washed and incubated with the TUNEL reaction mixture for 60 min at 37 °C. The slides were mounted with antifade mounting solution containing the nuclear dye 4′,6-diamidino-2-phenylindole (DAPI) (Sigma-Aldrich, Steinheim, Germany). Immunostaining intensity was analyzed using fluorescence microscopy (Olympus BX51).

### 2.11. Western blot

Liver samples were homogenized in radioimmunoprecipitation assay (RIPA) buffer (Polytron homogenizer, Kinematica, Lucerne, Switzerland). Proteins were isolated, separated by sodium dodecyl sulfate-polyacrylamide gel electrophoresis (SDS-PAGE) and blotted onto the a polyvinylidene fluoride (PVDF) membrane (Roche Diagnostics GmbH, Mannheim, Germany) as described previously.^27^ Membranes were washed and blocked (Roche Diagnostics GmbH, Mannheim, Germany), followed by incubation with antibodies against caspase-9 (ab185719), 4-hydroxynonenal (4-HNE, ab46545), and hem oxygenase-1 (HO-1, ab13243) (Abcam, Cambridge, UK). Primary antibodies to extracellular regulated kinase 1/2 (ERK1/2) (#4695), phosphorylated ERK1/2 (*p*-ERK1/2 Thr202/Tyr204, #4370), c-Jun N-terminal kinase 1/2 (JNK1/2) (#9252), phosphorylated JNK1/2 (*p*-JNK1/2 Thr183/Tyr185, #4668), p38 (#8690), phosphorylated p38 (p-p38 Thr180/Tyr182, #4511), cleaved caspase-3 (#9661), and microtubule-associated protein 1 light chain 3 beta (LC3B)-I/II (#2775) were from Cell Signaling Technologies (Beverly, MA, USA), and GAPDH (HRP-60004) was obtained from Proteintech, (Rosemont, IL, USA). Secondary antibodies, horseradish peroxidase (HRP)-conjugated goat polyclonal anti-mouse IgG (ab79023) and HRP-conjugated goat polyclonal anti-rabbit IgG (ab6721) were obtained from Abcam. After washing, the membranes were exposed to a chemiluminescent substrate (SignalFire Elite ECL, Cell Signaling Technologies, Beverly, MA, USA) and scanned using C-DiGit® Blot Scanner (LI-COR Biosciences, Lincoln, NE, USA). The intensity of the bands was analyzed using the computer image analysis software ImageJ.

### 2.12. Statistical analysis

Data were analyzed using StatSoft STATISTICA version 13.1. Differences between groups were assessed using one-way ANOVA and Tukey's posthoc test. Values are expressed as means ± SD. Differences were considered statistically significant at *P* < 0.05.

## 3. Results and discussion

### 3.1. BER-loaded polymersomes (BER-PS)

Previously, we demonstrated the hepatoprotective activity of BER in CCl_4_-intoxicated mice.^6^ To increase the bioavailability of BER at the site of ROS-mediated tissue injury, we developed BER-loaded PS based on HPMA as the hydrophilic block and a ROS-responsive PbAPE block. When loaded with the chemotherapeutic drug doxorubicin (DOX), these PS showed a decrease in tumor growth and prolonged animal survival in mice bearing EL4 T cell lymphoma with reduced side effects compared to free DOX. Such selective, site-specific action, highlights their great potential in environments with high ROS levels.^9^ BER-loaded polymersomes were prepared following our previously published protocols,^9^ which ensured the production of well-defined polymersomes using the microfluidics approach and ROS-responsive block copolymer.

**Fig. 1:**
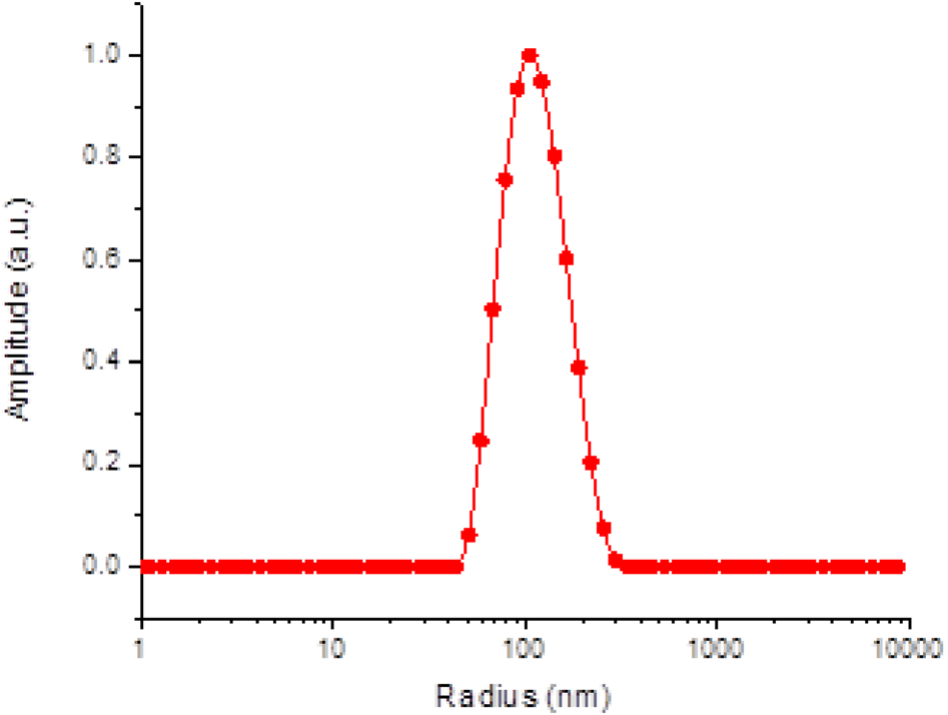
Distribution of sizes for BER-loaded PS.

DLS analysis (Fig. 1) revealed the formation of polymeric self-assemblies (PS) with uniform sizes. These assemblies exhibit reasonable homogeneity, as indicated by a polydispersity index (PDI) of less than 0.15 (Table 1), which can be attributed, at least in part, to the precision of the microfluidic-assisted manufacturing approach employed.^9,28^

**Table 1:** Structural characteristics of berberine-loaded PS determined *via* light scattering measurements

	*R*_H_ (nm)^a^	PDI^a^	ζ (mv)^b^
PHPMA_37_-*b*-PbAPE_42_	117.8	0.119	−12.5

a Measured by DLS.

b Measured by ELS.

**Fig. 2:**
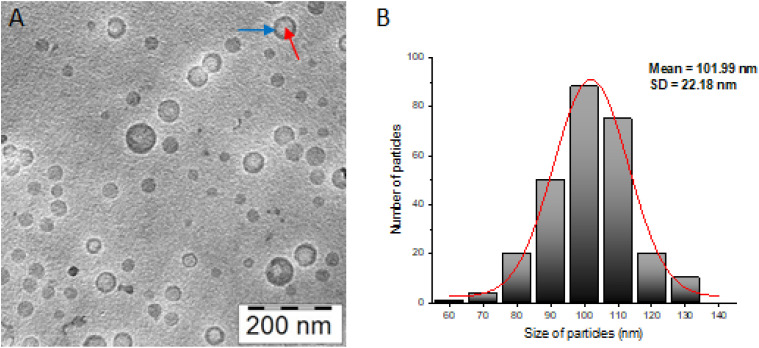
Cryo-TEM image of BER-loaded polymersomes (BER-PS); blue arrow shows polymeric bilayer, and red arrow indicates an aqueous core (A). Corresponding size distribution histogram calculated using ImageJ software (B).

The produced polymer colloids demonstrated notable stability, with no evidence of nanoparticle aggregation or significant changes in particle size or size dispersity. The surface charge (ζ-potential) of the self-assemblies was found to be slightly negative (Table 1). Given the nonionizable nature of the PHPMA shells, nearly neutral surfaces are expected. However, partial charge partitioning within the polymer shells typically results in a slightly negative surface charge, a characteristic commonly observed in such assemblies.^9,28–30^ Cryo-TEM imaging confirmed the successful formation of polymersomes, revealing well-defined spherical nanostructures with a distinct polymeric bilayer encapsulating an aqueous core (Fig. 2A). The vesicles displayed circular shapes with a darker rim and lighter interior, characteristic of unilamellar vesicular morphology. Image analysis was performed using ImageJ software to quantify the size distribution and further assess the morphology. The images were calibrated based on the scale bar and only intact and well-resolved particles were included in the analysis. The size distribution obtained from ImageJ was consistent with the DLS measurements, validating the uniformity and stability of the prepared polymersomes (Fig. 2B). These results confirmed the successful synthesis of nanoscale vesicles with controlled morphology and dimensions.

**Fig. 3:**
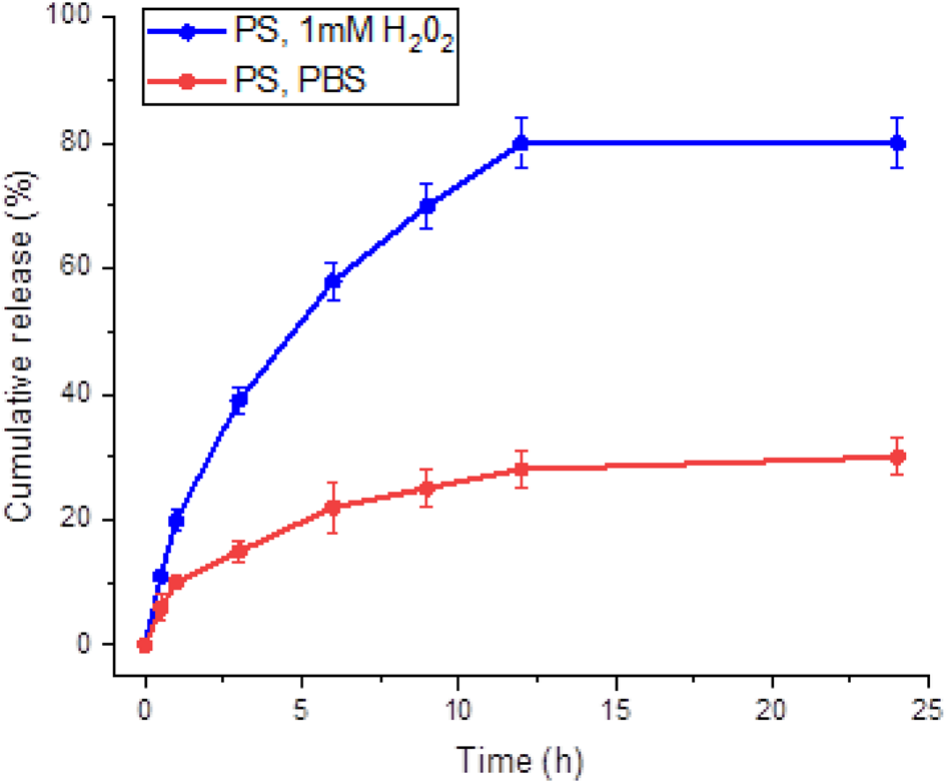
Release of berberine from BER-loaded PS in PBS and PBS containing 1 mM H_2_O_2_.

The release of berberine encapsulated within the synthesized polymersomes was expected to be influenced by their responsiveness to reactive oxygen species (ROS). To investigate this, *in vitro* release studies were conducted over 24 h in both PBS and PBS containing 1 mM H_2_O_2_, simulating a ROS-rich environment.^31,32^ The release rate of berberine was found to be approximately two times higher in the ROS-rich environment (Fig. 3, blue circles) compared to PBS alone (Fig. 3, red lines), suggesting that the polymersomes are responsive to oxidative stress.

Previously, we demonstrated that our ROS-responsive block copolymers are indeed ROS sensitive and that the *in vivo* therapeutic activity of loaded polymersomes can be attributed to their content.^9^ In the SI (Fig. S1), we included the results of *in vitro* analysis of the release of berberine from BER-loaded ROS-nonresponsive PS in PBS and PBS containing 1 mM H_2_O_2_. These data show a lower release of BER from ROS-nonresponsive PS compared to ROS-sensitive PS, suggesting that the effect is due to ROS sensitivity.

### 3.2. Serum biochemistry and body weight

**Fig. 4:**
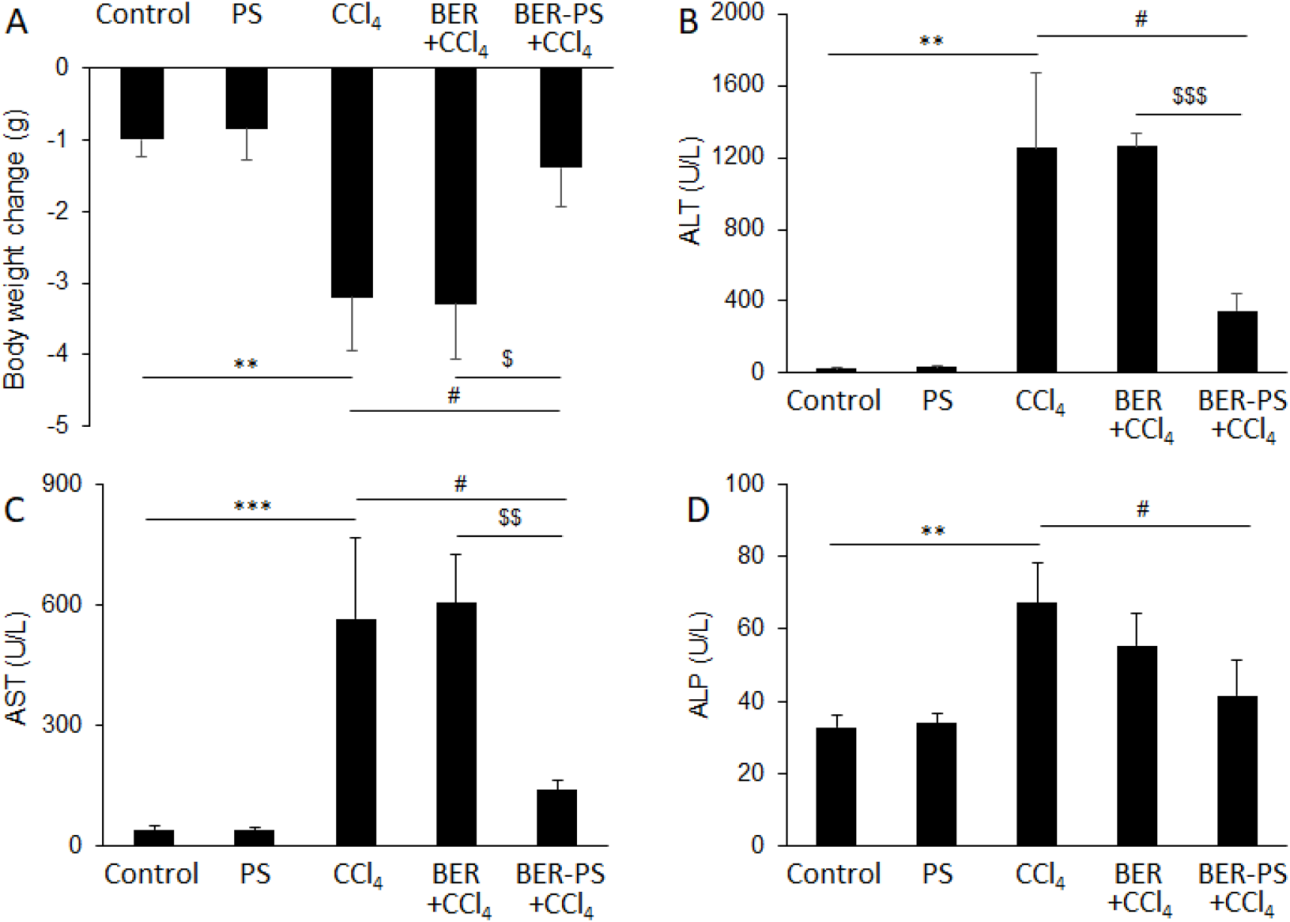
Body weight change and serum markers of liver injury. Mice were treated with vehicle (A), polimersomes (PS) (B), carbon tetrachloride (CCl4) (C), CCl_4_ + berberine (BER) (D), and CCl_4_ + BER-PS (E). Each value represents the mean ± SD for 6 mice. ** *p* < 0.01, *** *p* < 0.001 CCl_4_ compared to control; ^#^*p* < 0.05 CCl_4_ compared to BER + CCl_4_ and BER-PS + CCl_4_; ^$^*p* < 0.05, ^$$^*p* < 0.01, ^$$$^*p* < 0.001 BER + CCl_4_ compared to BER-PS + CCl_4_.

CCl_4_-intoxication in animals is a widely used experimental model of ROS-induced liver injury and a screening method for developing novel drugs and therapeutic approaches. Cellular damage caused by free radicals generated during CCl_4_ metabolism results in altered plasma membrane permeability or even destruction of the cell, resulting in the release of cytosolic content into circulation. Serum AST and ALT are well-known markers of liver injury and their levels increase gradually, reaching a maximum 24 h after CCl_4_ administration to mice.^33^ Treatment with CCl_4_ caused a slight decrease in mice body weight after CCl_4_ administration, suggesting animal distress (Fig. 4A).^34^ Biochemical analyses of serum AST, ALT, and ALP activities were performed to determine whether BER and BER-PS protected the liver from CCl_4_-induced injury. Serum hepatic enzyme levels were significantly increased after CCl_4_ intoxication (Fig. 4B and 4C). Treatment with BER-PS attenuated these changes compared with BER, preserving the structural integrity of the hepatic tissue.

### 3.3. Histopathology

**Fig. 5:**
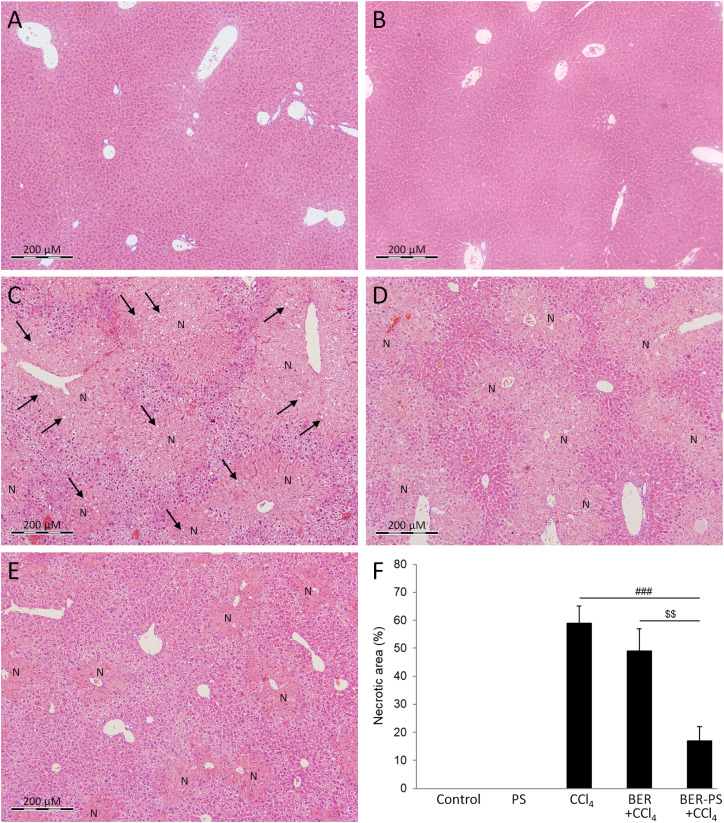
Hematoxylin and eosin-stained microphotographs of liver tissue. Mice were treated with vehicle (A), polimersomes (PS) (B), carbon tetrachloride (CCl_4_) (C), CCl_4_ + berberine (BER) (D), and CCl_4_ + BER-PS (E). Original magnification × 100. Letter “N” denotes necrotic areas, arrows show microsteatosis. Liver injury score (F). Each value represents the mean ± SD for 6 mice. Representative images from at least 10 random fields (×100). ^###^*p* < 0.001 CCl_4_ compared to BER + CCl_4_ and BER-PS + CCl_4_; ^$$^*p* < 0.01 BER + CCl_4_ compared to BER-PS + CCl_4_.

ROS hyperproduction during CCl_4_ metabolism affects the structure and function of cellular components.^35^ Livers from control mice (Fig. 5A) and PS-treated mice (Fig. 5B) exhibited normal tissue architecture. In the CCl_4_ group, large areas of centrilobular necrosis and microvesicular steatosis were found two days after CCl_4_ administration (Fig. 5C). Histopathological changes, including necrosis and steatosis, were significantly attenuated by BER-PS compared to free BER. BER did not exert a notable hepatoprotective effects and massive necrotic areas were still present (Fig. 5D). However, BER-PS markedly reduced hepatocellular damage (Fig. 5E), indicating minimal necrosis of the hepatic tissue (Fig. 5F).

### 3.4. Expression of oxidative stress, apoptosis, and autophagy markers

**Fig. 6:**
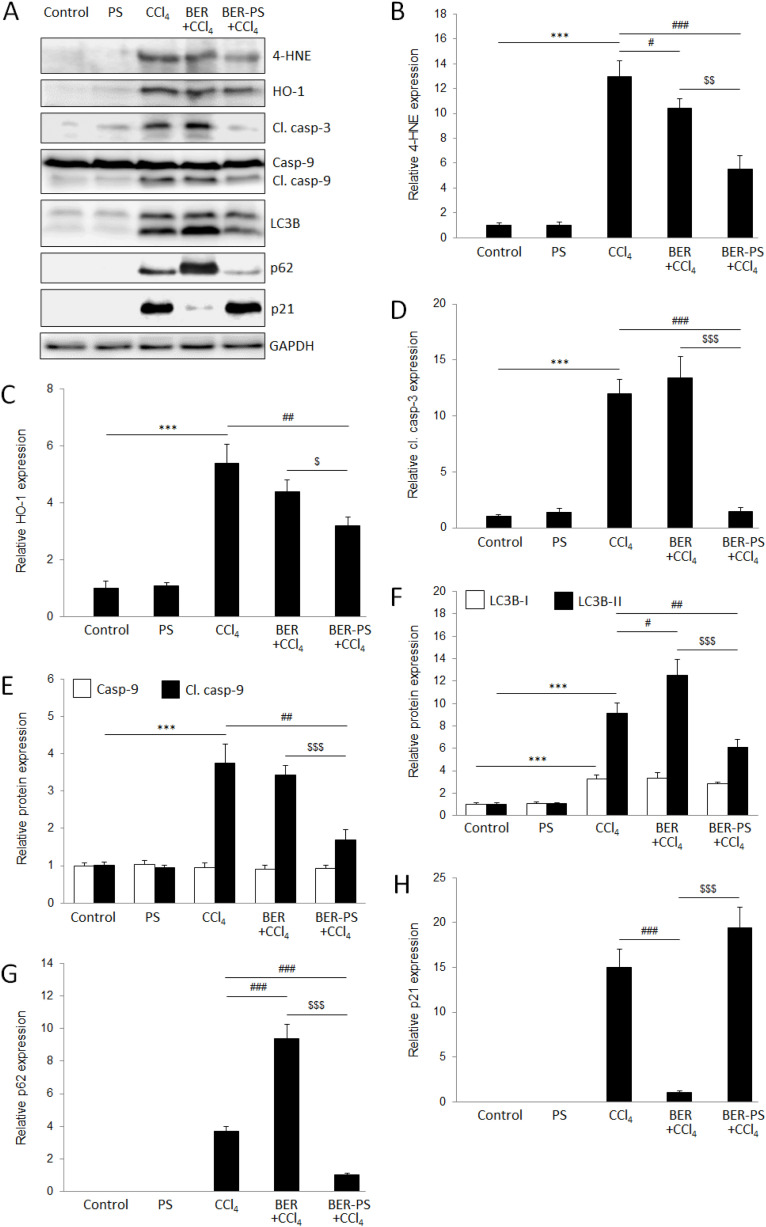
Western blot analysis of oxidative stress, apoptosis, and autophagy markers in the liver (A). Mice were treated with vehicle, polimersomes (PS), carbon tetrachloride (CCl_4_), CCl_4_ + berberine (BER), and CCl_4_ + BER-PS. Semiquantification of the expression of 4-hydroxynonenal (4-HNE) (B), hem oxygenase-1 (HO-1) (C), cleaved caspase-3 (D), caspase-9 (E), microtubule-associated protein 1 light chain 3 beta (LC3B)-I/II (F), p62 (G), and p21 (H). Each value represents the mean ± SD for 6 mice. *** *p* < 0.001 CCl_4_ compared to control; ^#^*p* < 0.05, ^##^*p* < 0.01, ^###^*p* < 0.001 CCl_4_ compared to BER + CCl_4_ and BER-PS + CCl_4_; ^$^*p* < 0.05, ^$$^*p* < 0.01, ^$$$^*p* < 0.001 BER + CCl_4_ compared to BER-PS + CCl_4_.

**Fig. 7:**
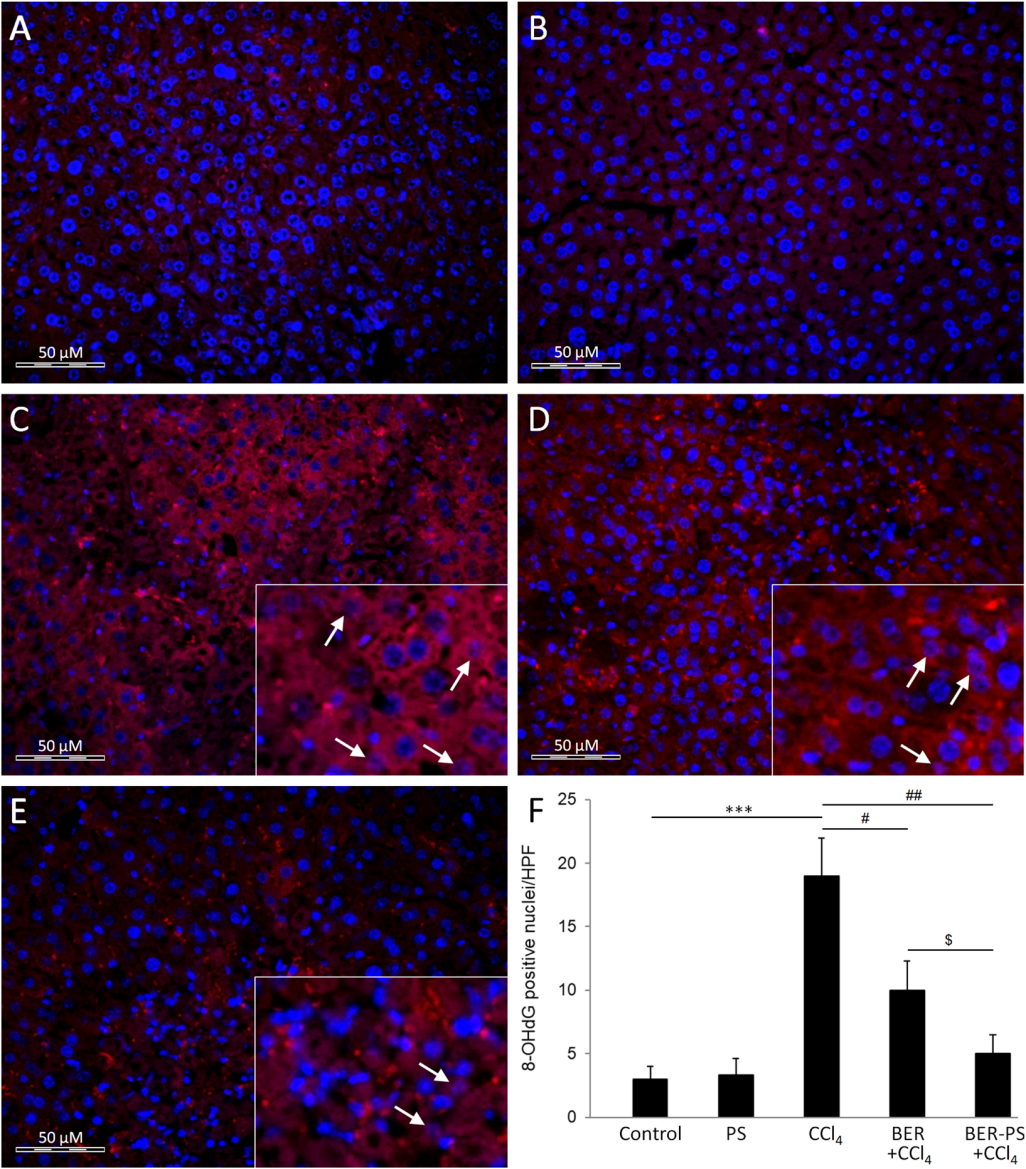
Immunofluorescence analysis of 8-hydroxy-2′-deoxyguanosine (8-OHdG) in the liver of mice treated with vehicle (A), polimersomes (PS) (B), carbon tetrachloride (CCl_4_) (C), CCl_4_ + berberine (BER) (D), and CCl_4_ + BER-PS (E). Measurement of the intensity of 8-OHdG immunopositive staining (F). Each value represents the mean ± SD for 6 mice. Representative images from at least 10 high-power fields (×400). Insets: enlarged view of 8-OHdG immunopositive cells (arrows). *** *p* < 0.001 CCl_4_ compared to control; ^#^*p* < 0.05, ^##^*p* < 0.01 CCl_4_ compared to BER + CCl_4_ and BER-PS + CCl_4_; ^$^*p* < 0.05 BER + CCl_4_ compared to BER-PS + CCl_4_.

Western blot analysis showed increased expression of oxidative stress, apoptosis, and autophagy proteins (Fig. 6A). Increased expression and activity of antioxidant enzymes and damage to cellular lipids are hallmarks of CCl_4_-induced liver damage.^36,37^ The liver, as a major site of increased ROS production due to CCl_4_ metabolism, could benefit from targeted, site-specific delivery of active compounds carried by ROS-sensitive PS. In the current study, the expression of the lipid peroxidation oxidative stress marker 4-HNE (Fig. 6B) and the antioxidant enzyme HO-1 (Fig. 6C) significantly increased in the CCl_4_ group compared to the control group and PS-treated animals. Treatment of CCl_4_-intoxicated mice with BER-PS resulted in a marked reduction of oxidative stress markers compared to the BER-treated group. Hepatic oxidative DNA damage was assessed by immunostaining with an 8-OHdG antibody. The liver of control and PS-treated mice showed a low presence of 8-OHdG (Fig. 7A and B), strong nuclear 8-OHdG immunoreactivity was detected in the liver parenchyma injured with CCl_4_ (Fig. 7C). However, 8-OHdG immunoreactivity was reduced by BER treatment (Fig. 7D) and was markedly suppressed by BER-PS (Fig. 7E). These results are in agreement with those presented in Fig. 3, showing increased BER release from BER-PS in the ROS-enriched medium.

**Fig. 8:**
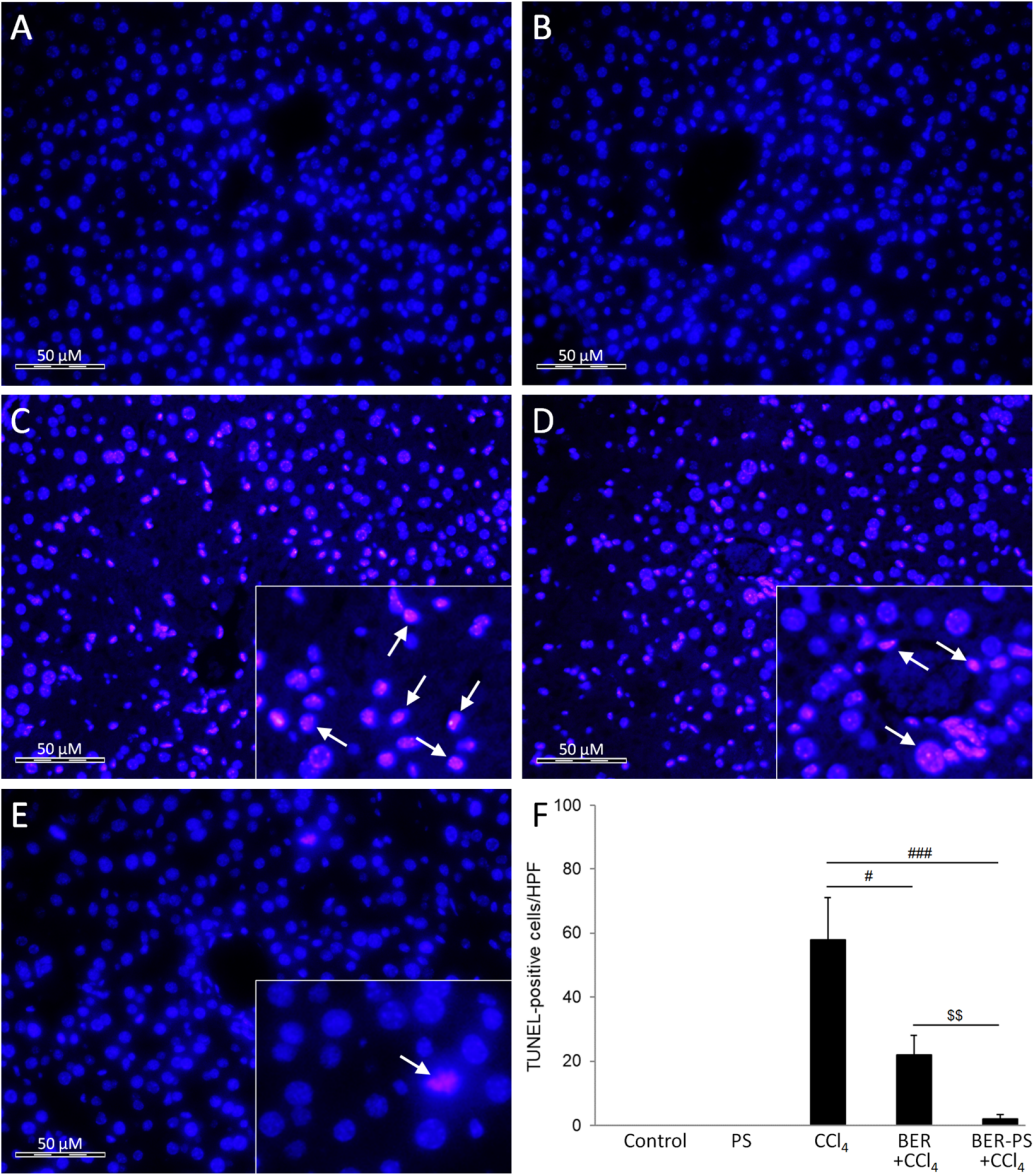
TUNEL assay of the liver of mice treated with vehicle (A), polimersomes (PS) (B), carbon tetrachloride (CCl_4_) (C), CCl_4_ + berberine (BER) (D), and CCl_4_ + BER-PS (E). Number of TUNEL-positive hepatocytes (F). Each value represents the mean ± SD for 6 mice. Representative images from at least 10 high-power fields (×400). Insets: enlarged view of TUNEL-positive cells (arrows). ^#^*p* < 0.05, ^###^*p* < 0.001 CCl_4_ compared to BER + CCl_4_ and BER-PS + CCl_4_; ^$$^*p* < 0.01 BER + CCl_4_ compared to BER-PS + CCl_4_.

Free radicals generated during CCl_4_ metabolism also induce apoptosis in liver tissue.^32,38^ Apoptosis can be triggered *via* two different pathways, intrinsic and extrinsic. Intracellular stress triggers intrinsic apoptosis, controlled by the B-cell lymphoma 2 protein family, leading to the activation of caspase-9 and, finally, executioner caspases.^39^ Previously, BER was shown to inhibit apoptosis in CCl_4_-treated rats.^40^ In the current study, the expression of apoptotic markers cleaved caspase-3 (Fig. 6D) and caspase 9 (Fig. 6E) was increased in mice treated with CCl_4_. Administration of BER did not result in significant amelioration of apoptosis, however, BER-PS significantly reduced caspase expression in the liver. These results were confirmed using the TUNEL assay (Fig. 8). The control and PS-treated mice did not show signs of TUNEL-positive cells (Fig. 8A and B). The CCl_4_-treated group showed marked TUNEL positivity (Fig. 8C), which was reduced by BER treatment (Fig. 8D) and markedly suppressed in the BER-PS group (Fig. 8E). This suggests that BER-PS was more effective in suppressing of apoptotic cell death in the liver, which is consistent with the histological findings.

Free radical-mediated liver injury may also result in activation of autophagy.^33^ Autophagy is an intracellular lysosome-mediated digestion system that removes unnecessary or dysfunctional cellular components, such as protein aggregates or damaged organelles.^41^ The conversion of LC3B from an unconjugated form (LC3B-I) to a phosphatidylethanolamine (PE)-conjugated form (LC3B-II) is crucial for autophagosome formation. p62 is a multifunctional adaptor protein critically involved in autophagy, acting as an autophagy receptor that delivers substrates to autophagosomes for degradation, becoming degraded during the process.^42^ BER has been recently shown to significantly enhance autophagic flux and restore autolysosomal function in ischemic neuronal injury.^43^ BER also promoted autophagy and the maturation and expression of lysosomes in dextran sulfate sodium-induced ulcerative colitis.^44^ On the other hand, BER protected the myocardium against ischemia/reperfusion injury by inhibiting excessive autophagy.^45^ In the current study, treatment with CCl_4_ led to the induction of both p62 and LC3B-II when compared to the control and PS groups (Fig. 6F and G). This is in accordance with previous research,^33^ which has shown that CCl_4_-induced acute liver injury inhibits the later stages of autophagy flux by impairing autophagosome-lysosome fusion, resulting in LC3B-II and p62 accumulation. Nevertheless, the reduction in LC3B-II and p62 expression in BER-PS treatment compared to CCl_4_ and BER treatments suggested amelioration of autophagy, reflecting reduced liver tissue injury.

The expression of p21 (Fig. 6H) was opposite of p62, which was highly expressed in the CCl_4_ and BER-PS groups, compared to the control and PS groups, and reduced with BER treatment. Interestingly, Jung and coworkers showed that cell cycle inhibitor p21 upregulation may result in autophagy-mediated downregulation of p62.^46^ These findings are in agreement with our current results, suggesting that the higher hepatoprotective activity of BER-PS compared to BER was achieved through p21-mediated suppression of p62.

Nanostructures, such as polymeric nanoparticles, are also involved in modulation of cellular processes, such as autophagy and apoptosis. Exposure to nanoparticles often results in the blockade or disruption of the autophagic process.^47^ Therefore, the impact of the nanocarrier in BER-PS must be considered. However, control mice treated with PS did not show significant changes in oxidative stress, inflammatory response, apoptosis, or autophagy compared to the untreated controls. Nevertheless, the differences in the expression of autophagy proteins in the BER-PS-treated group could be attributed to PS, which markedly suppressed autophagy compared to free BER.

### 3.5. Expression of key signaling pathways

**Fig. 9:**
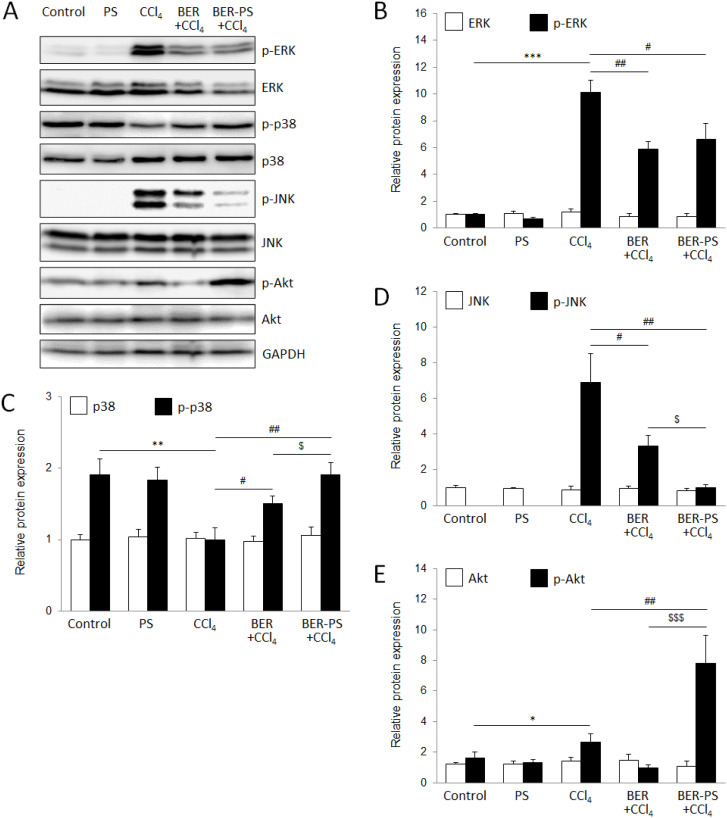
Western blot analysis of mitogen-activated protein kinases and Akt expression in the liver (A). Mice were treated with vehicle, polimersomes (PS), carbon tetrachloride (CCl_4_), CCl_4_ + berberine (BER), and CCl_4_ + BER-PS. Semiquantification of the expression of extracellular signal-regulated kinase (ERK1/2) and activated ERK1/2 (*p*-ERK1/2) (B), p38 and activated p38 (p-p38) (C), c-Jun N-terminal kinase (JNK1/2) and activated JNK1/2 (*p*-JNK1/2) (D), and Akt and activated Akt (*p*-Akt) (E). Each value represents the mean ± SD for 6 mice. * *p* < 0.05, ** *p* < 0.01, *** *p* < 0.001 CCl_4_ compared to control; ^#^*p* < 0.05, ^##^*p* < 0.01 CCl_4_ compared to BER + CCl_4_ and BER-PS + CCl_4_; ^$^*p* < 0.05, ^$$$^*p* < 0.001 BER + CCl_4_ compared to BER-PS + CCl_4_.

To investigate the molecular mechanism underlying the hepatoprotection of BER and BER-PS, we measured the expression of Akt, ERK1/2, JNK1/2, and p38 MAPK using western blot analysis (Fig. 8A). The phosphoinositide 3-kinase (PI3K)/Akt pathway plays a central role in regulating autophagy. Akt modulates autophagy initiation in response to increased ROS levels.^48^ A moderate increase in ROS levels appears to lead to a greater occurrence of autophagy, whereas higher levels of ROS reduce the incidence of autophagy. In the current study, BER reduced activation of Akt induced by CCl_4_ (Fig. 9E), which coincided with the upregulation of the autophagy markers LC3B-II and p62.^49,50^ However, BER-PS strongly activated Akt, resulting in the suppression of autophagy initiation.

The MAPK pathway is a well-known intracellular signal transduction cascade commonly involved in cell proliferation and survival and it is frequently found up-regulated during ROS-mediated hepatic injury.^33,38,51^ The MAPK pathway can promote pro-apoptotic processes, however, MAPKs, particularly JNK and p38, are also involved in anti-apoptotic mechanisms, suggesting a cell context and cell type-specific manner in the regulation of apoptosis.^52^ Moreover, in CCl_4_-induced liver injury, the activation of MAPKs was strongly time-dependent.^33^ Our results showed significantly increased expression of *p*-ERK1/2 (Fig. 9B) and *p*-JNK1/2 (Fig. 9D) 48 h after CCl_4_-intoxication, with a concomitant decrease in p-p38 (Fig. 9C). Both BER and BER-PS treatments markedly decreased ERK1/2 phosphorylation, whereas *p*-JNK1/2 expression was more suppressed by BER-PS, suggesting their pro-apoptotic role. The upregulation of p-p38 by BER, particularly BER-PS, compared to the CCl_4_ group, coincided with the reduced expression of *p*-JNK1/2. Consistent with the other results, BER-PS more effectively promoted a protective effect against CCl_4_-induced liver injury than BER, through the mechanism involving increased p38 activation, with concomitant suppression of JNK1/2 activation. Previously, it has been suggested that the p38 pathway can negatively regulate JNK activity.^51^ Chemical inhibition of p38 or deletion of the p38 strongly increased the activation of JNK *in vitro* and *in vivo* models and the cell fate seems to be sealed when JNK activity surpassed a threshold level.^53^

### 3.6. NF-κB and TNF-α expression

**Fig. 10:**
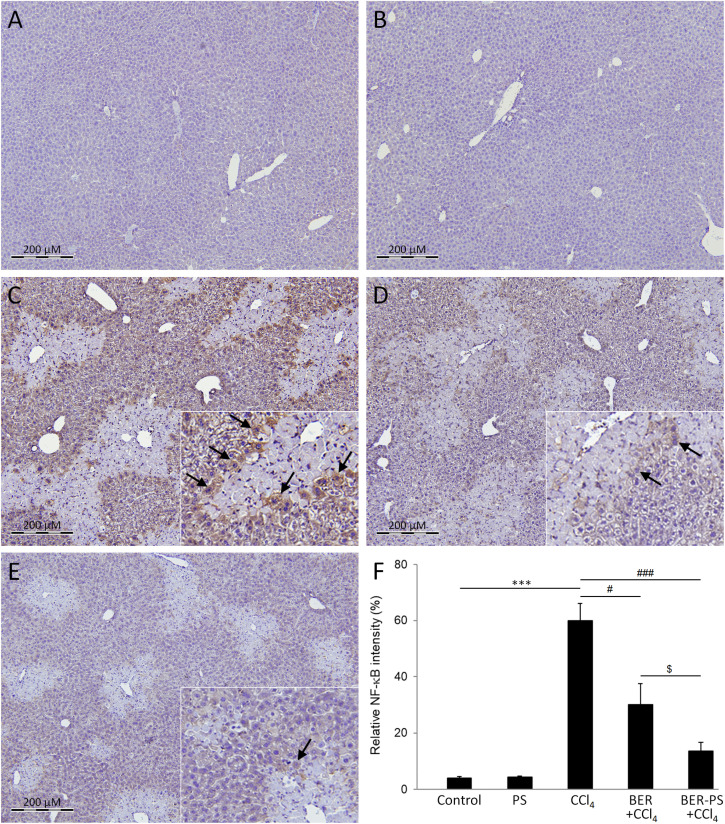
Immunohistochemical staining for nuclear factor kappaB (NF-κB) in the liver. Mice were treated with vehicle (A), polimersomes (PS) (B), carbon tetrachloride (CCl_4_) (C), CCl_4_ + berberine (BER) (D), and CCl_4_ + BER-PS (E). Measurement of NF-κB immunopositive staining intensity (F). Each value represents the mean ± SD for 6 mice. Representative images from at least 10 random fields (×100). Insets: enlarged view of NF-κB-immunopositive cells (arrows). *** *p* < 0.001 CCl_4_ compared to control; ^#^*p* < 0.05, ^##^*p* < 0.01, ^###^*p* < 0.001 CCl_4_ compared to BER + CCl_4_ and BER-PS + CCl_4_; ^$^*p* < 0.05 BER + CCl_4_ compared to BER-PS + CCl_4_.

**Fig. 11:**
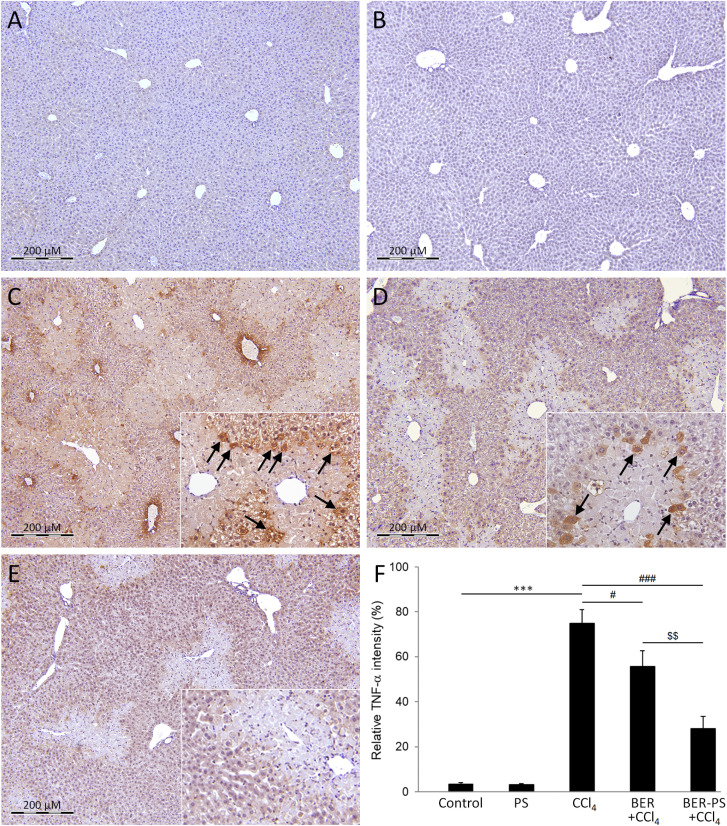
Immunohistochemical staining for tumor necrosis factor-alpha (TNF-α) in the liver. Mice were treated with vehicle (A), polimersomes (PS) (B), carbon tetrachloride (CCl_4_) (C), CCl_4_ + berberine (BER) (D), and CCl_4_ + BER-PS (E). Measurement of TNF-α immunopositive staining intensity (F). Each value represents the mean ± SD for 6 mice. Representative images from at least 10 random fields (×100). Insets: enlarged view of TNF-α-immunopositive cells (arrows). *** *p* < 0.001 CCl_4_ compared to control; ^#^*p* < 0.05, ^###^*p* < 0.001 CCl_4_ compared to BER + CCl_4_ and BER-PS + CCl_4_; ^$$^*p* < 0.01 BER + CCl_4_ compared to BER-PS + CCl_4_.

CCl_4_-intoxication also induces NF-κB expression in hepatocytes,^27^ resulting in activation of pro-inflammatory cytokines, such as TNF-α.^38^ TNF-α is a multifunctional cytokine produced by hepatic cells, Kupffer cells, and immune cells infiltrating the liver after liver injury but can also invade the liver *via* systemic circulation.^54^ TNF-α can induce various biological responses, including hepatocyte apoptosis and liver inflammation.^55^ The expression of hepatic NF-κB and TNF-α was measured using immunohistochemistry. The livers of control mice and mice treated with PS did not show significant NF-κB immunopositivity (Fig. 10A and B). As expected, NF-κB expression was induced in the CCl_4_ group, mainly in the remaining hepatocytes (Fig. 10C). NF-κB immunoreactivity was still observed in mice that received BER (Fig. 10D) and BER-PS (Fig. 10E), limited to the boundary between the healthy tissue and necrotic areas. Notably, the BER-PS-treated group showed lower NF-κB expression than the BER-treated group. The expression of TNF-α was negligible in the control groups (Fig. 11A and B). In the CCl_4_ group, high TNF-α immunopositivity indicated increased production of a major pro-inflammatory cytokine (Fig. 11C). Treatment with BER (Fig. 11D) slightly reduced and BER-PS (Fig. 11E) markedly suppressed its production in liver tissue. The decrease in NF-κB and TNF-α expression following BER-PS treatment suggests a more effective amelioration of CCl_4_-induced pro-inflammatory response in the liver compared to BER.

## 4. Conclusions

**Fig. 12:**
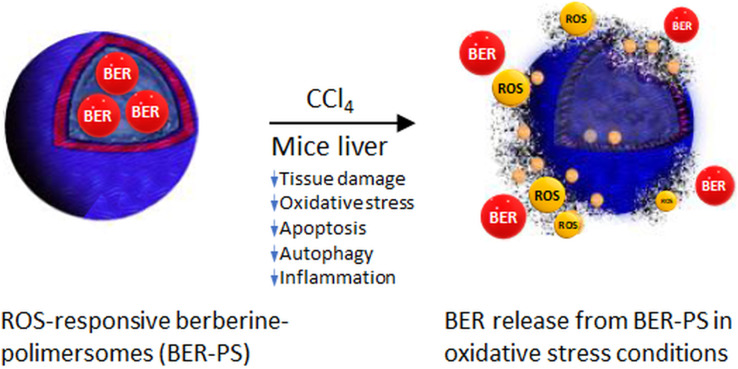
Release of berberine (BER) from berberine-polimersomes (BER-PS) in reactive oxygen species (ROS)-rich environment in the liver due to carbon tetrachloride (CCl_4_) metabolism, and the mechanism of BER-PS hepatoprotective activity against CCl_4_ liver injury.

The liver, as a major site of CCl_4_ biotransformation, is the site of increased ROS production. Our results suggest that ROS-responsive BER-PS has greater potential for the prevention of acute liver injury induced by CCl_4_ than free BER. Mechanistically, BER-PS efficiently suppressed oxidative stress and regulated key signaling pathways involved in the inflammatory response, apoptosis, and autophagy. The increased hepatoprotective activity of ROS-sensitive BER-PS in free radical environments is attributed to the specific targeting and enhanced delivery of the active compound to the site of injury. The study is summarized in Fig. 12.

## 5. Study limitations and future directions

The current study did not include nonresponsive PS. Nevertheless, our *in vitro* data (Fig. S1) suggest that nonresponsive PS have a low potential to release their cargo compared to ROS-responsive BER-PS, suggesting that the BER-PS effect is due to ROS-sensitivity.

While the PS did not show detectable toxicity in the control group, a more extensive evaluation of potential long-term side effects or accumulation of the nanocarriers should be conducted in the future.

In the future, thorough pharmacokinetic analyses, including tissue accumulation profiles of BER, should be performed to demonstrate the absorption and biodistribution of BER-PS.

Further studies are needed to confirm whether these results translate to human physiology. Additional models and clinical studies would strengthen the translational impact of this study.

## Author contributions

Iva Suman: investigation, formal analysis, supervision, writing – review & editing, funding acquisition. Damir Klepac: funding acquisition; methodology; project administration; supervision. Martina Vragović: investigation; methodology; visualization. Hrvoje Križan: investigation. Eliézer Jäger: resources; methodology; supervision. Alessandro Jäger: resources; methodology; supervision. Ewa Pavlova: investigation; methodology. Martin Hrubý: supervision; resources; funding acquisition. Robert Domitrović: conceptualization, methodology, formal analysis, resources, writing – original draft, project administration, funding acquisition.

## Conflicts of interest

There are no conflicts to declare.

## Supplementary Material

NA-008-D5NA00706B-s001

## Data Availability

The data supporting this article have been included as part of the supplementary information (SI). Supplementary information is available. See DOI: https://doi.org/10.1039/d5na00706b.
