# Supplementary material for: Targeted delivery of berberine via ROS-sensitive polymersomes enhances its hepatoprotective activity in CCl4-intoxicated mice
Source: Nanoscale Adv. 2025 Dec 3;8(2):595–611. doi: 10.1039/d5na00706b (PMC12687027; doi:10.1039/d5na00706b)
Supplement: NA-008-D5NA00706B-s001 [file NA-008-D5NA00706B-s001.pdf]

Supplementary Information (SI) for Nanoscale Advances.
This journal is © The Royal Society of Chemistry 2025

Supplementary Materials

Journal: Nanoscale Advances

Title: Targeted delivery of berberine via ROS-sensitive polymersomes enhances its hepatoprotective activity in CCl4-intoxicated mice

Iva Suman, Damir Klepac, Martina Vragović, Hrvoje Križan, Eliézer Jäger, Alessandro Jäger, Ewa Pavlova, Martin Hrubý, Robert Domitrović*

*Prof. Robert Domitrović, Ph. D., Department of Medical Chemistry, Biochemistry and Clinical Chemistry, Faculty of Medicine, University of Rijeka, B. Branchetta 20, 51000 Rijeka, Croatia. E-mail: robert.domitrovic@medri.uniri.hr.

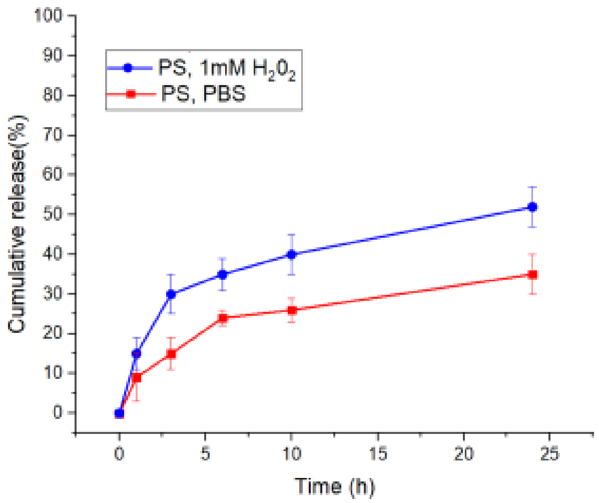

Fig. S1 Release of berberine from BER-loaded non-responsive PS in PBS and PBS containing 1 mM H_2O_2.

To compare the release of berberine, we prepared non-responsive polymersomes based on a non -responsive poly[N-(4-isopropylphenylacetamide)ethyl methacrylate] (PPPhA) block (DOI: 10.1021/acs.biomac.4c00282). The release rate of berberine in the presence of ROS-rich environment was approximately 50% higher compared to ROS-responsive PS.

The full uncropped blots images.

4-HNE

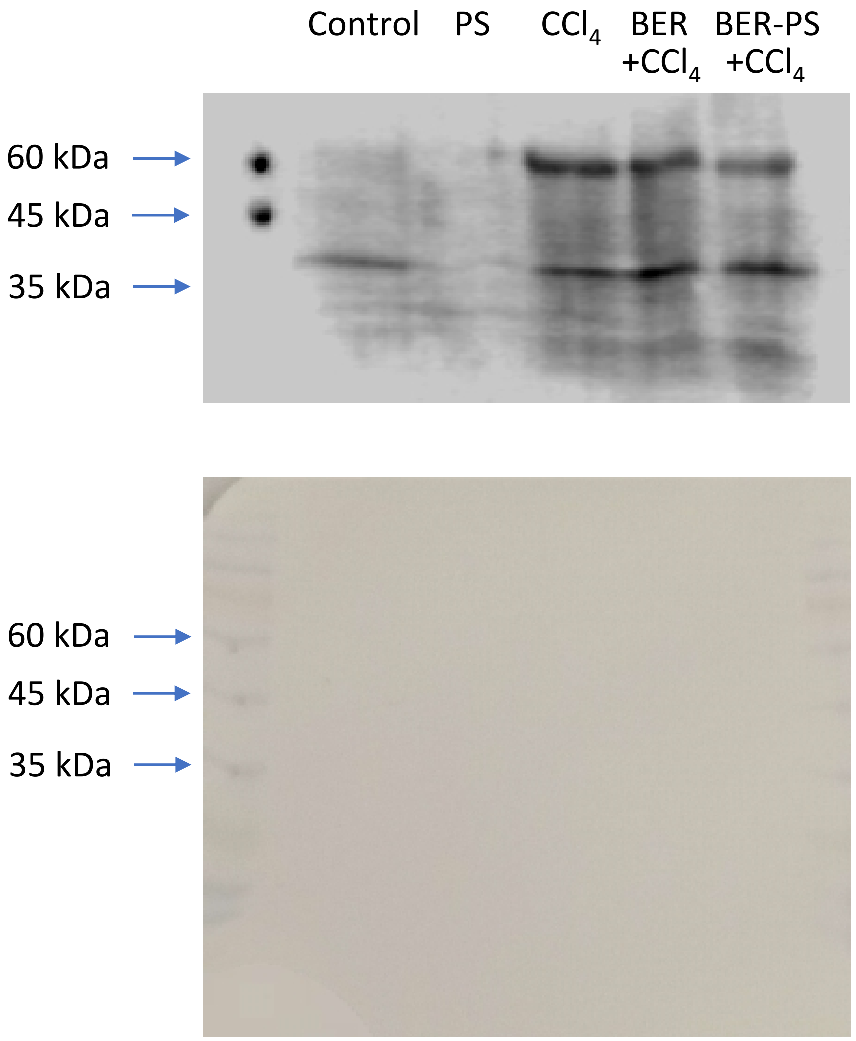

HO-1

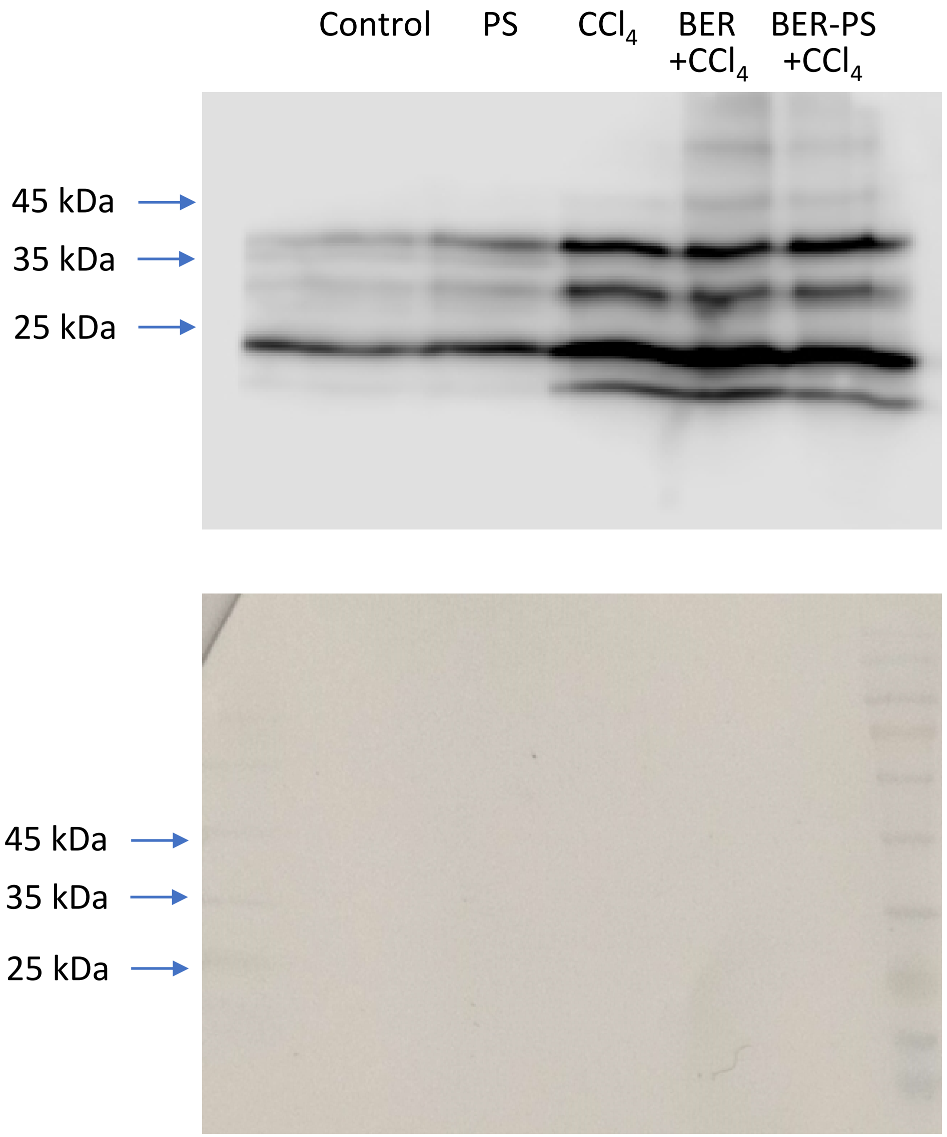
Control
PS
CCl4
BER +CCl4
BER-PS +CCl4
45 kDa
35 kDa
25 kDa
45 kDa
35 kDa
25 kDa

Casp 3

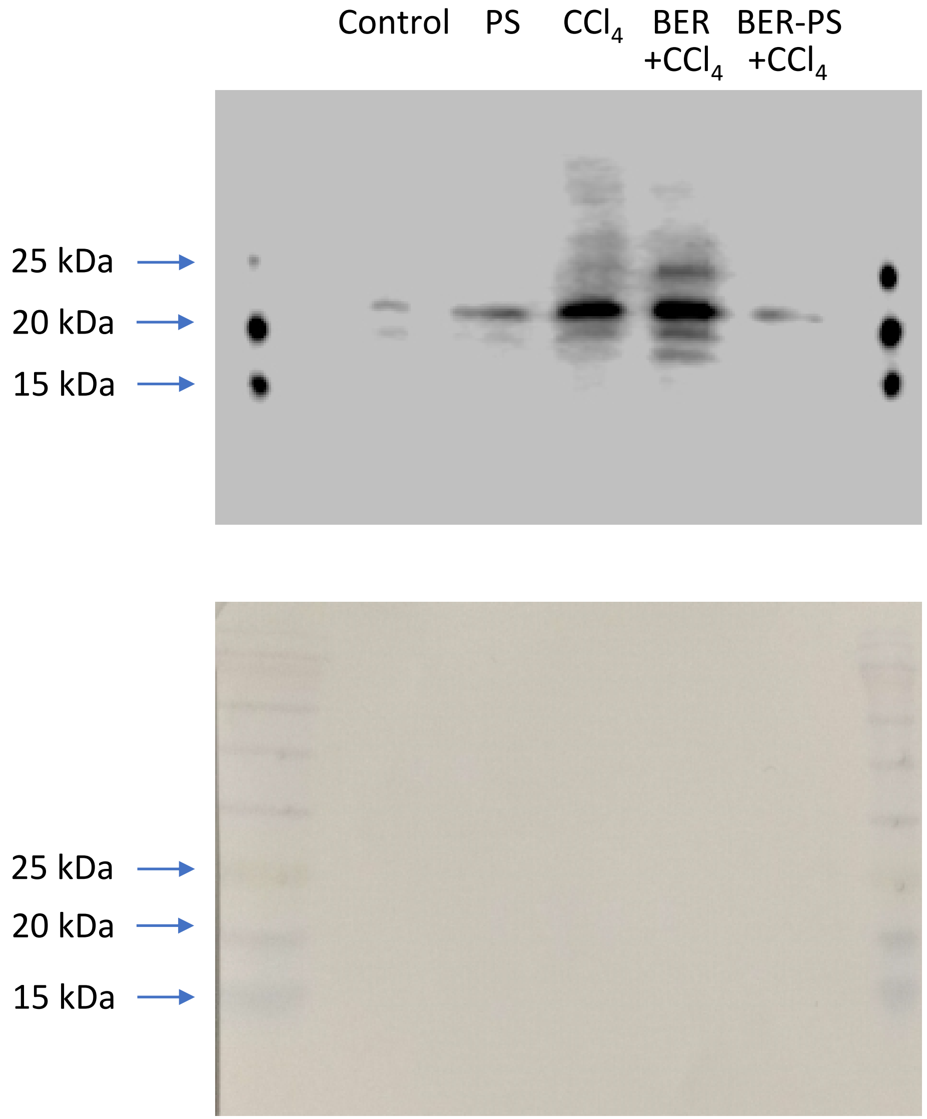
Control
PS
CCl4
BER +CCl4
BER-PS +CCl4
25 kDa
20 kDa
15 kDa
25 kDa
20 kDa
15 kDa

Casp 9, Cl. Casp 9.

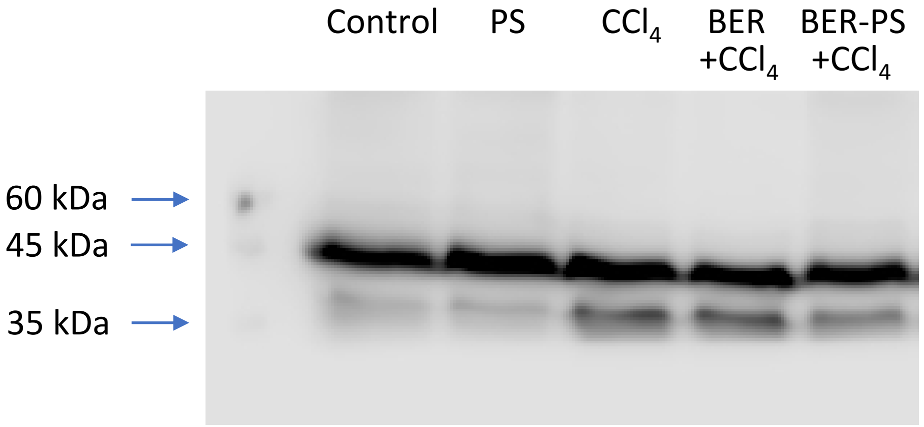

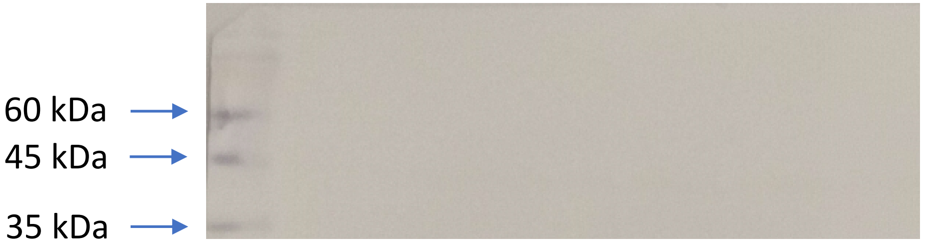

LC3

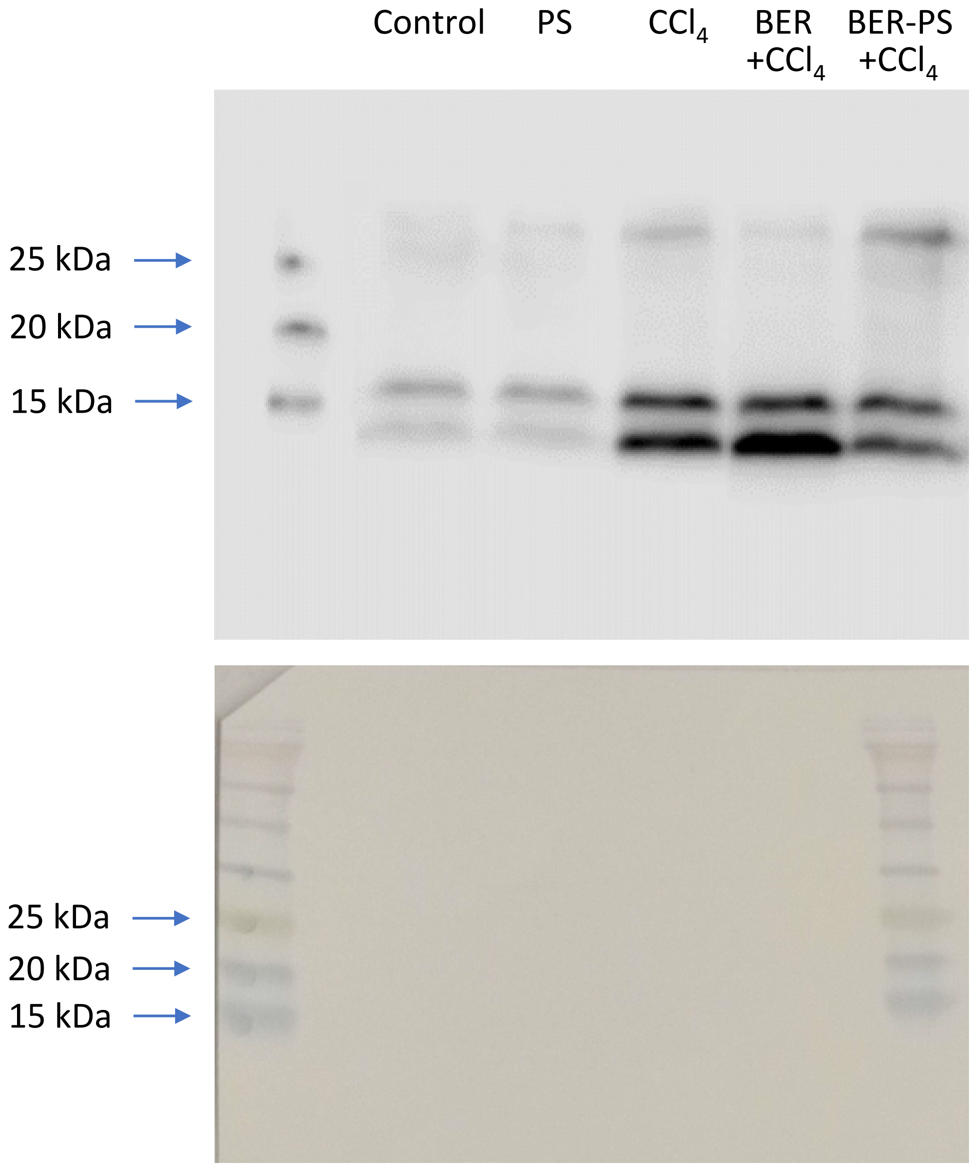
Control
PS
CCl4
BER +CCl4
BER-PS +CCl4
25 kDa
20 kDa
15 kDa
25 kDa
20 kDa
15 kDa

p62

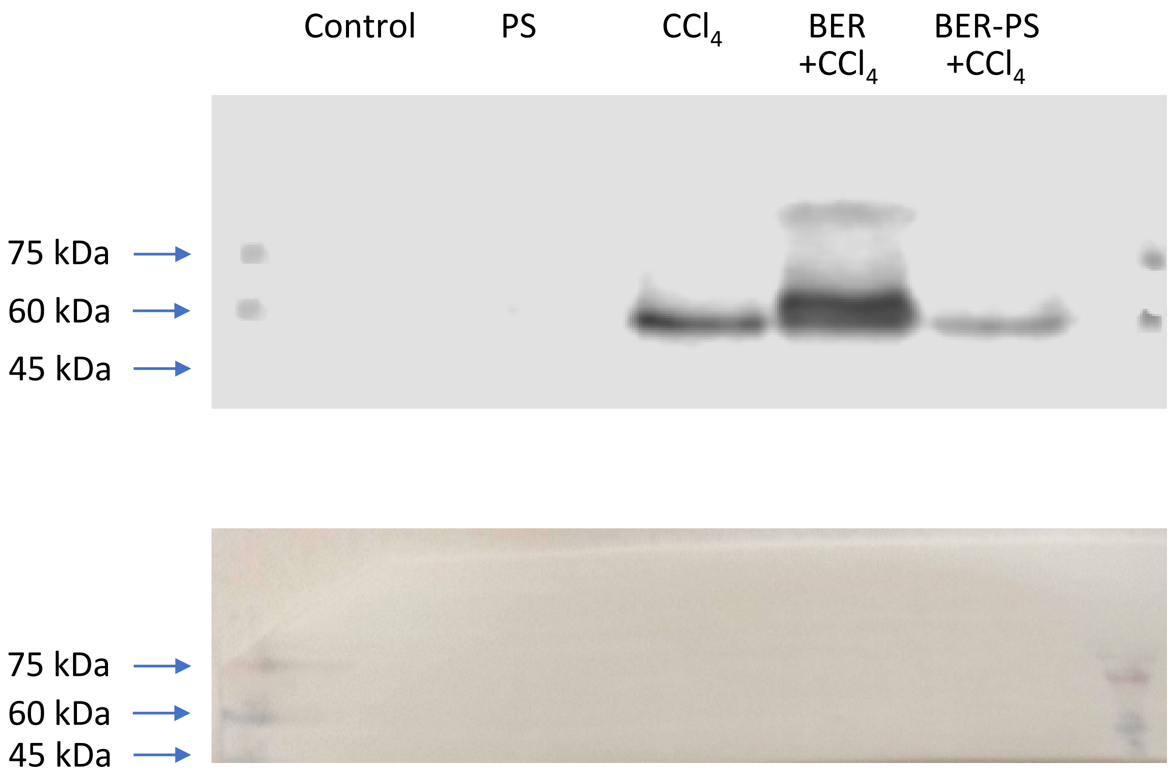
Control
PS
CCl_4
BER +CCl_4
BER-PS +CCl_4
75 kDa
60 kDa
45 kDa
75 kDa
60 kDa
45 kDa

p21

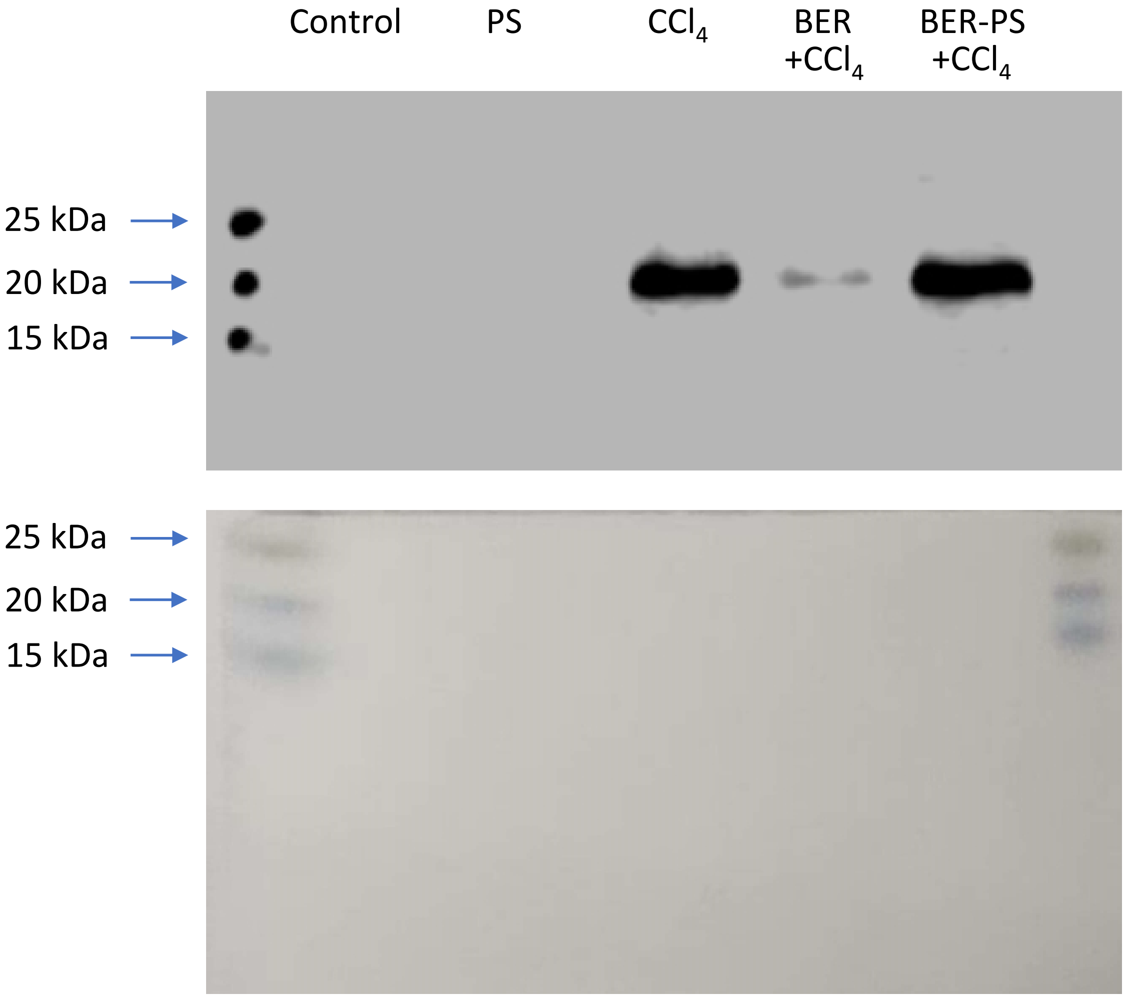
Control
PS
CCl4
BER +CCl4
BER-PS +CCl4
25 kDa
20 kDa
15 kDa
25 kDa
20 kDa
15 kDa

GAPDH

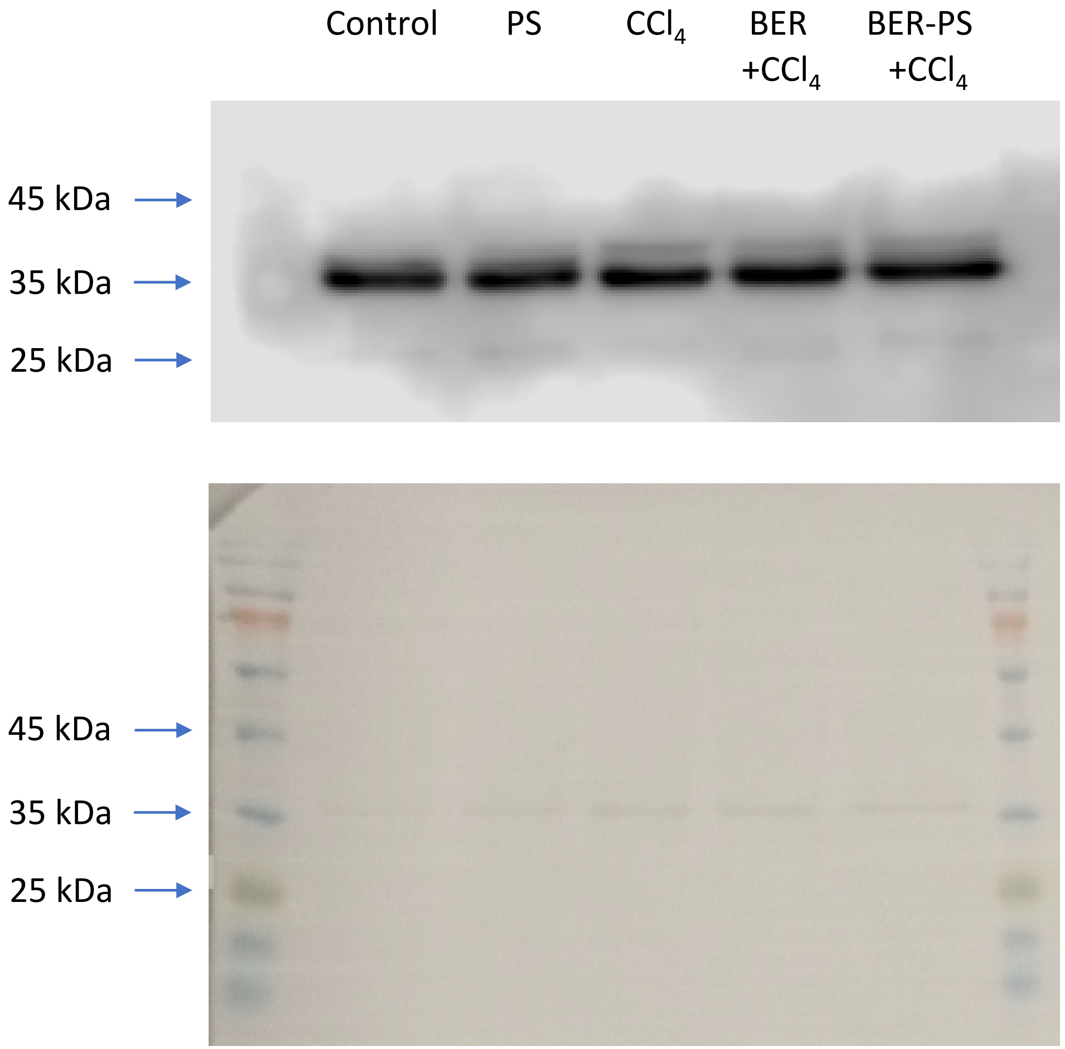
Control
PS
CCl_4
BER +CCl_4
BER-PS +CCl_4
45 kDa
35 kDa
25 kDa
45 kDa
35 kDa
25 kDa

p-ERK

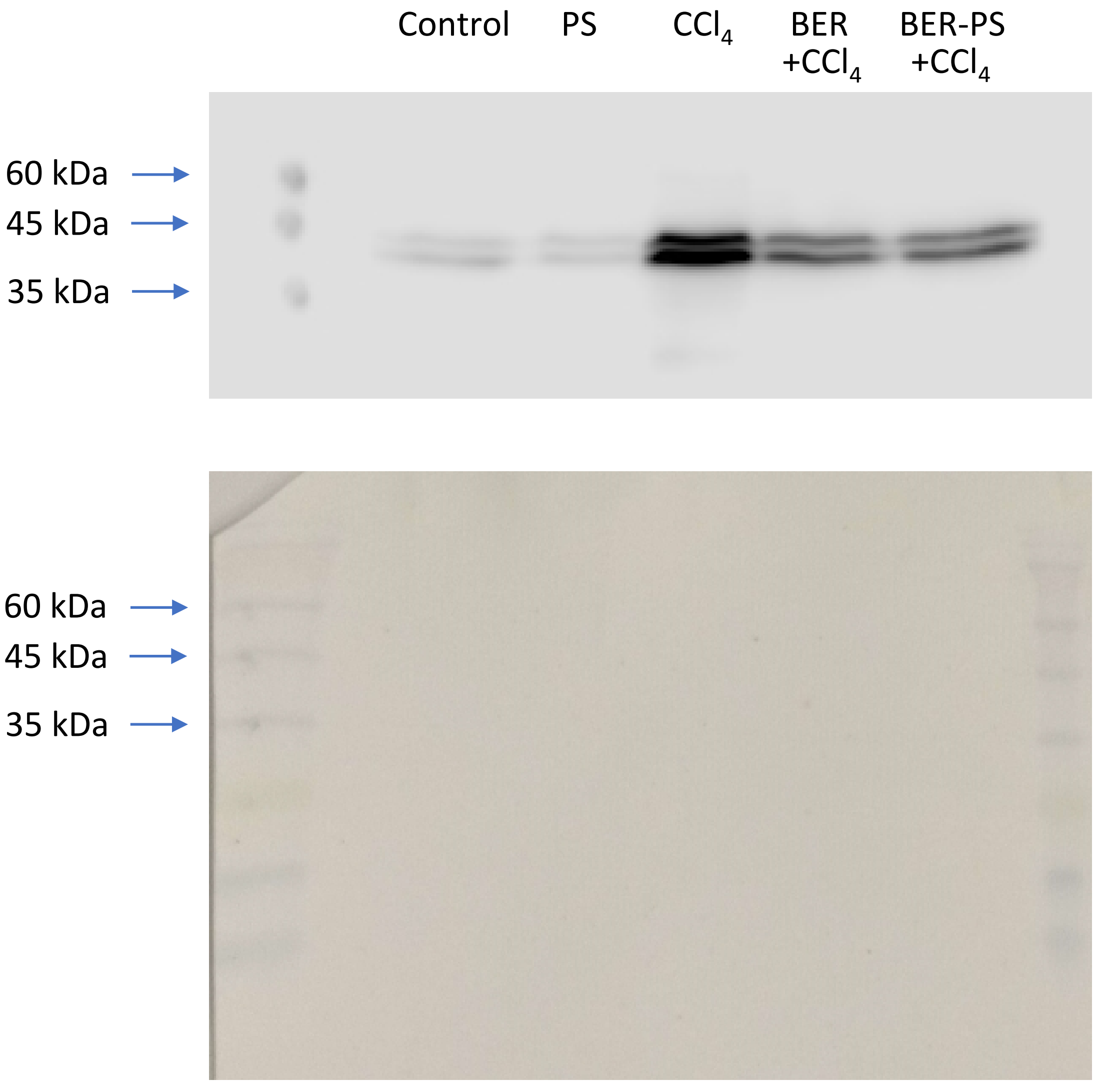
Control
PS
CCl4
BER +CCl4
BER-PS +CCl4
60 kDa
45 kDa
35 kDa
60 kDa
45 kDa
35 kDa

ERK

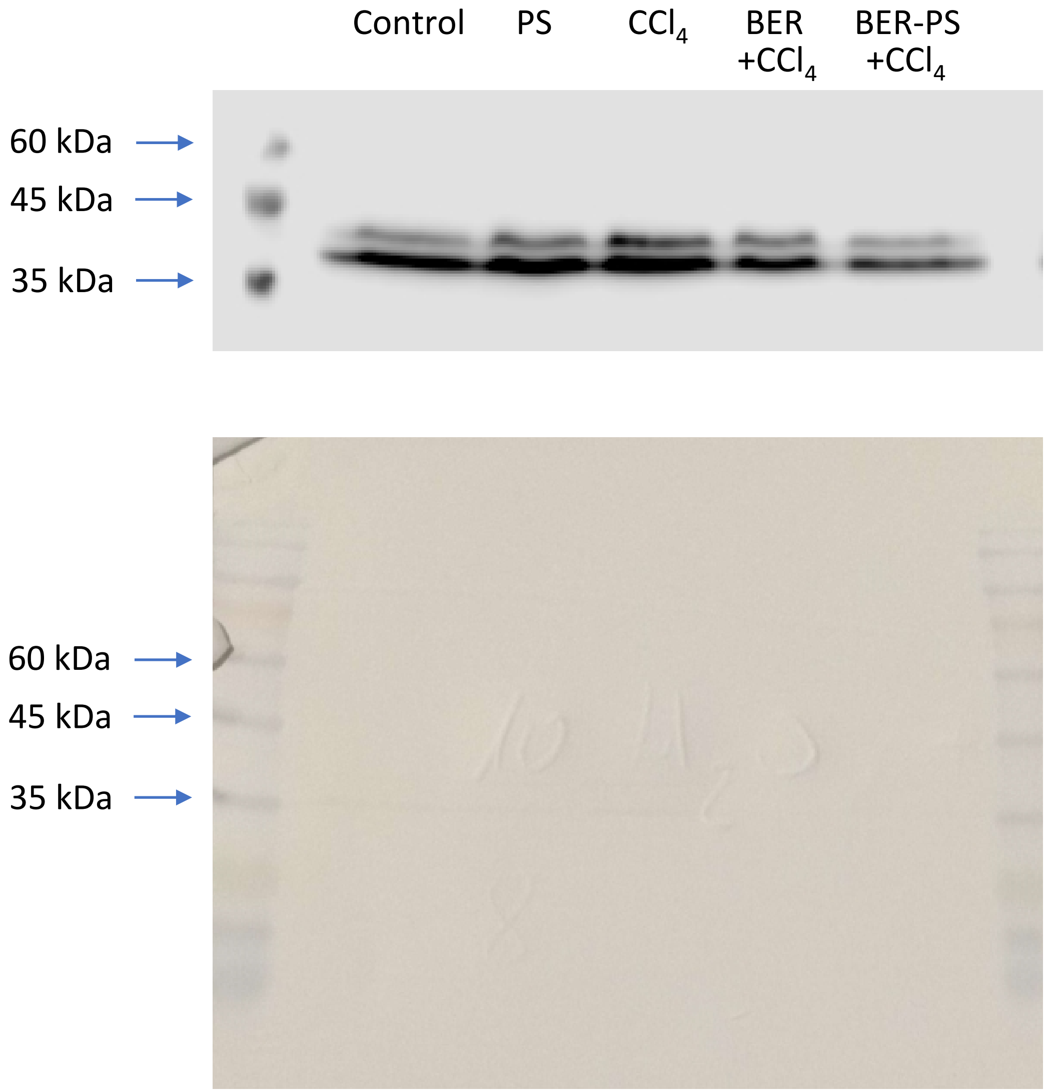
Control
PS
CCl_4
BER $+CCl_4$
BER-PS $+CCl_4$
60 kDa
45 kDa
35 kDa
60 kDa
45 kDa
35 kDa

p-p38

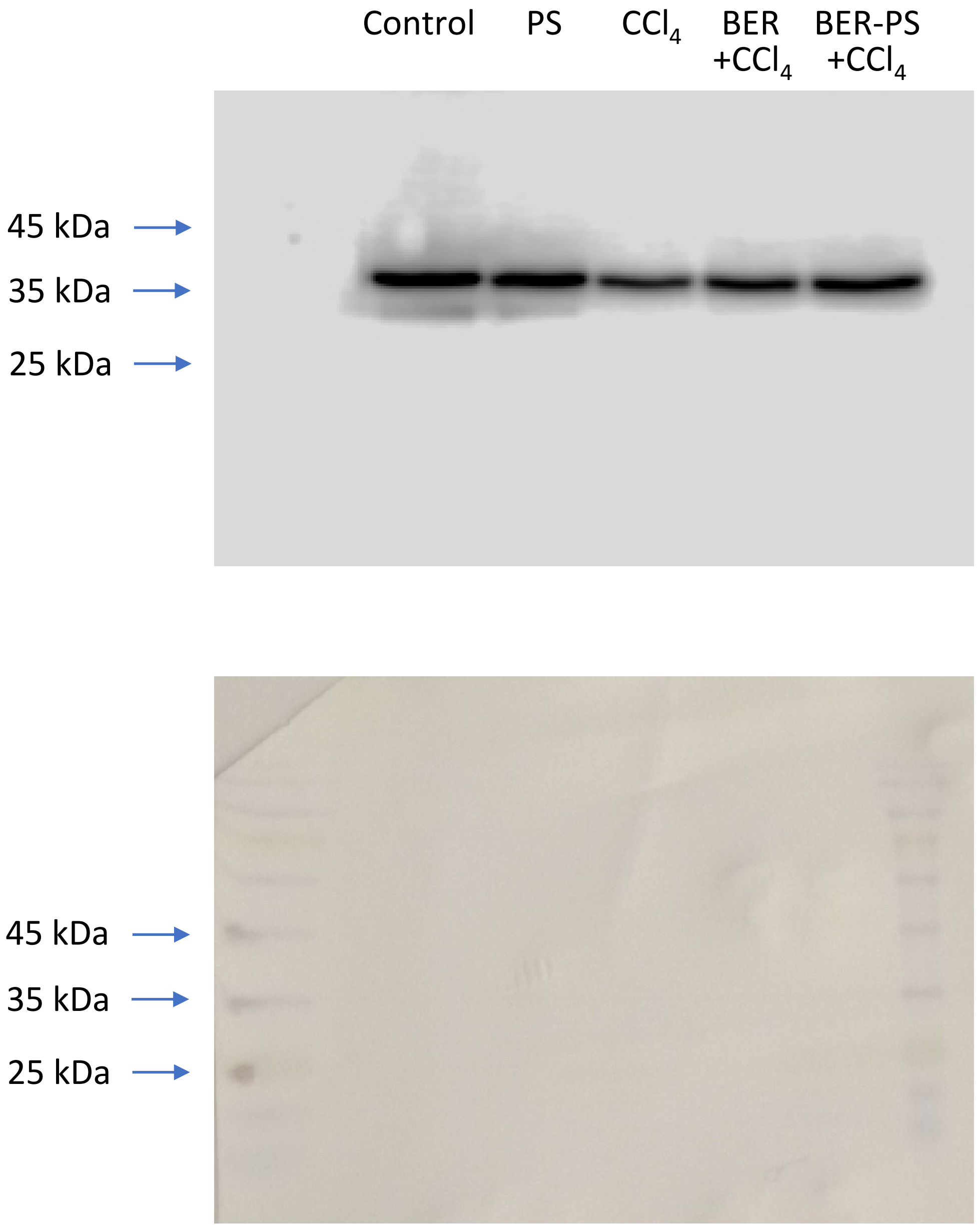
Control
PS
CCl_4
BER +CCl_4
BER-PS +CCl_4
45 kDa
35 kDa
25 kDa
45 kDa
35 kDa
25 kDa

p38

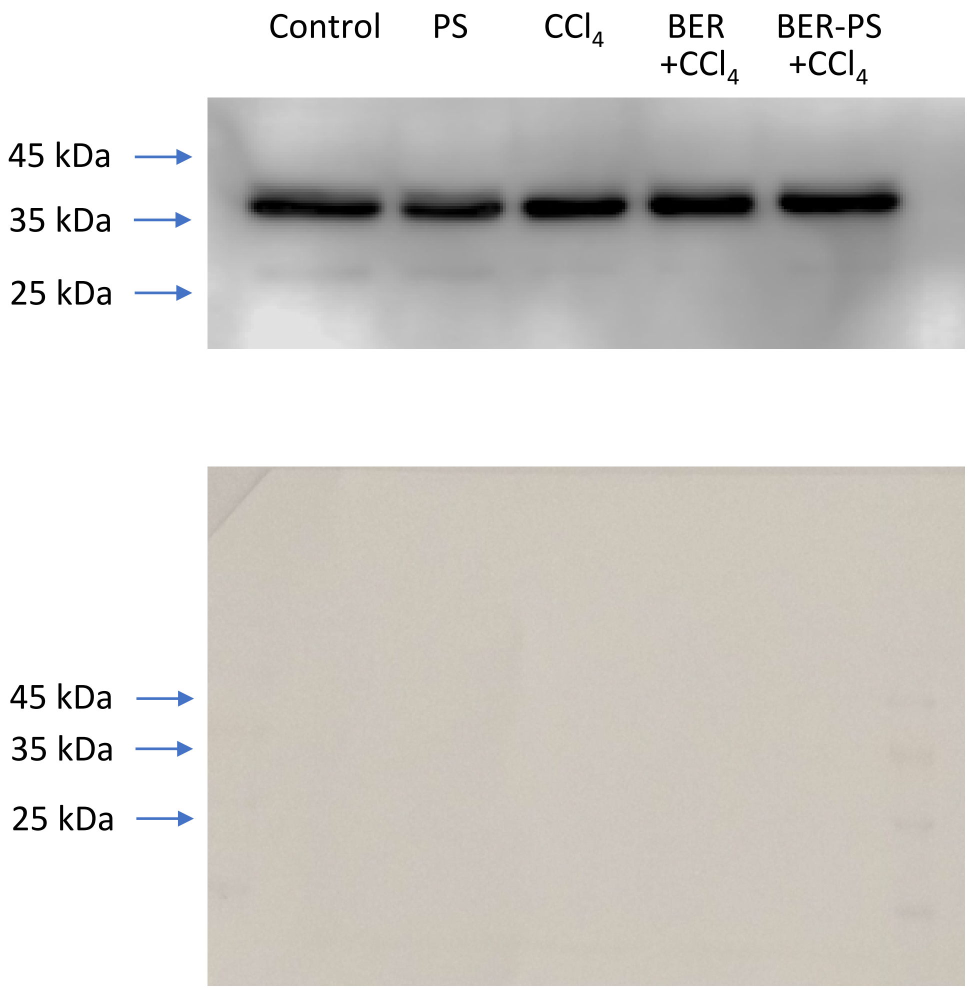
Control
PS
CCl4
BER +CCl4
BER-PS +CCl4
45 kDa
35 kDa
25 kDa
45 kDa
35 kDa
25 kDa

p-JNK

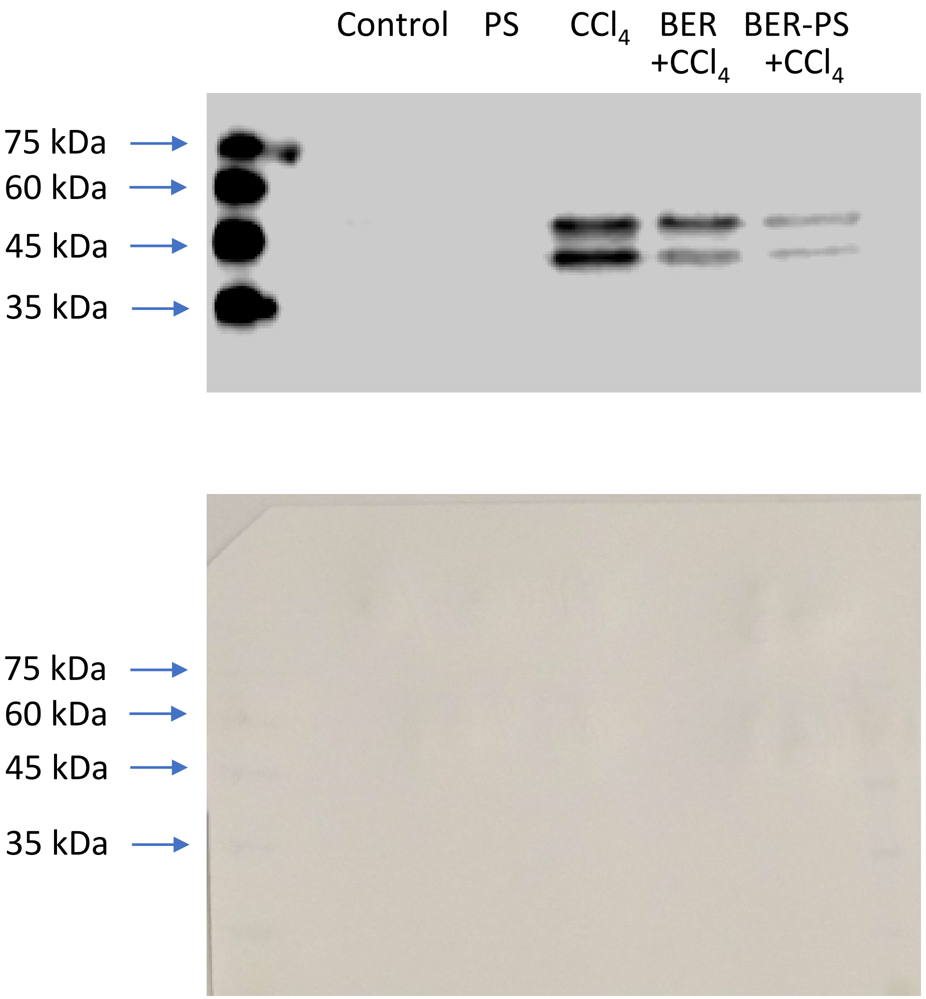
Control
PS
CCl4
BER +CCl4
BER-PS +CCl4
75 kDa
60 kDa
45 kDa
35 kDa
75 kDa
60 kDa
45 kDa
35 kDa

JNK

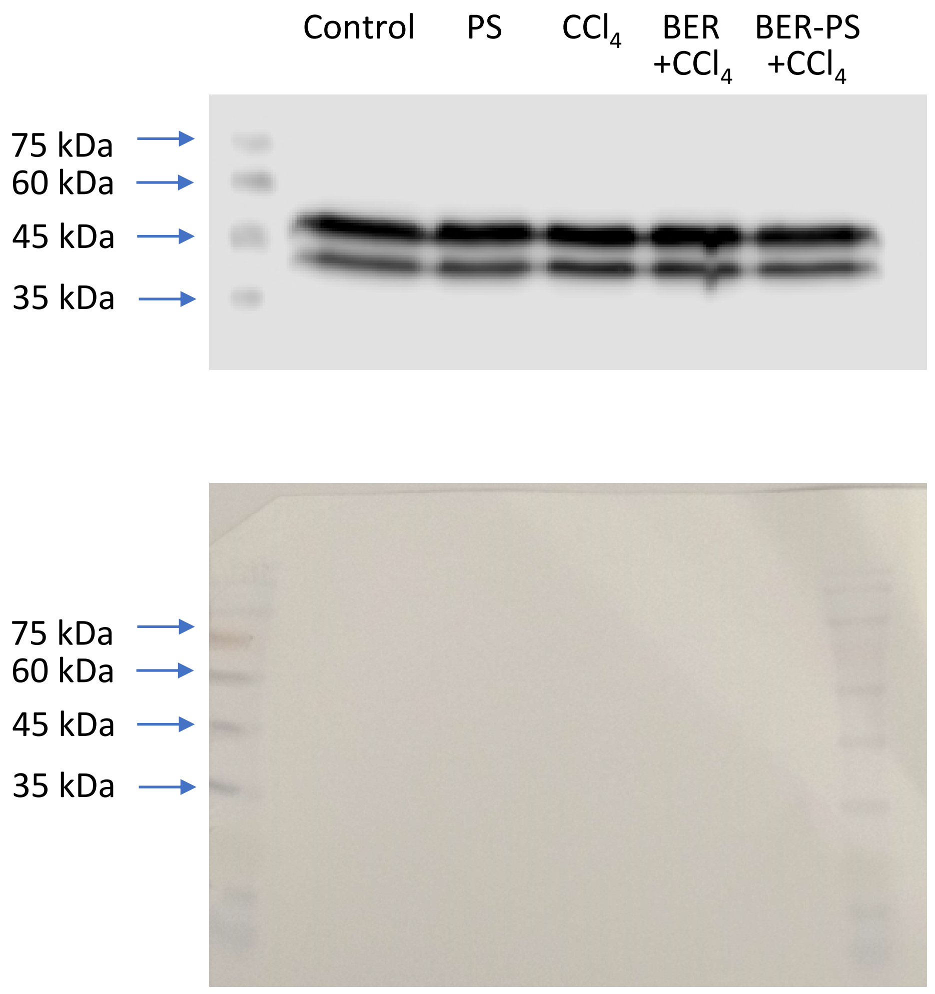

Control
PS
CCl_4
BER +CCl_4
BER-PS +CCl_4
75 kDa
60 kDa
45 kDa
35 kDa
75 kDa
60 kDa
45 kDa
35 kDa

P-Akt

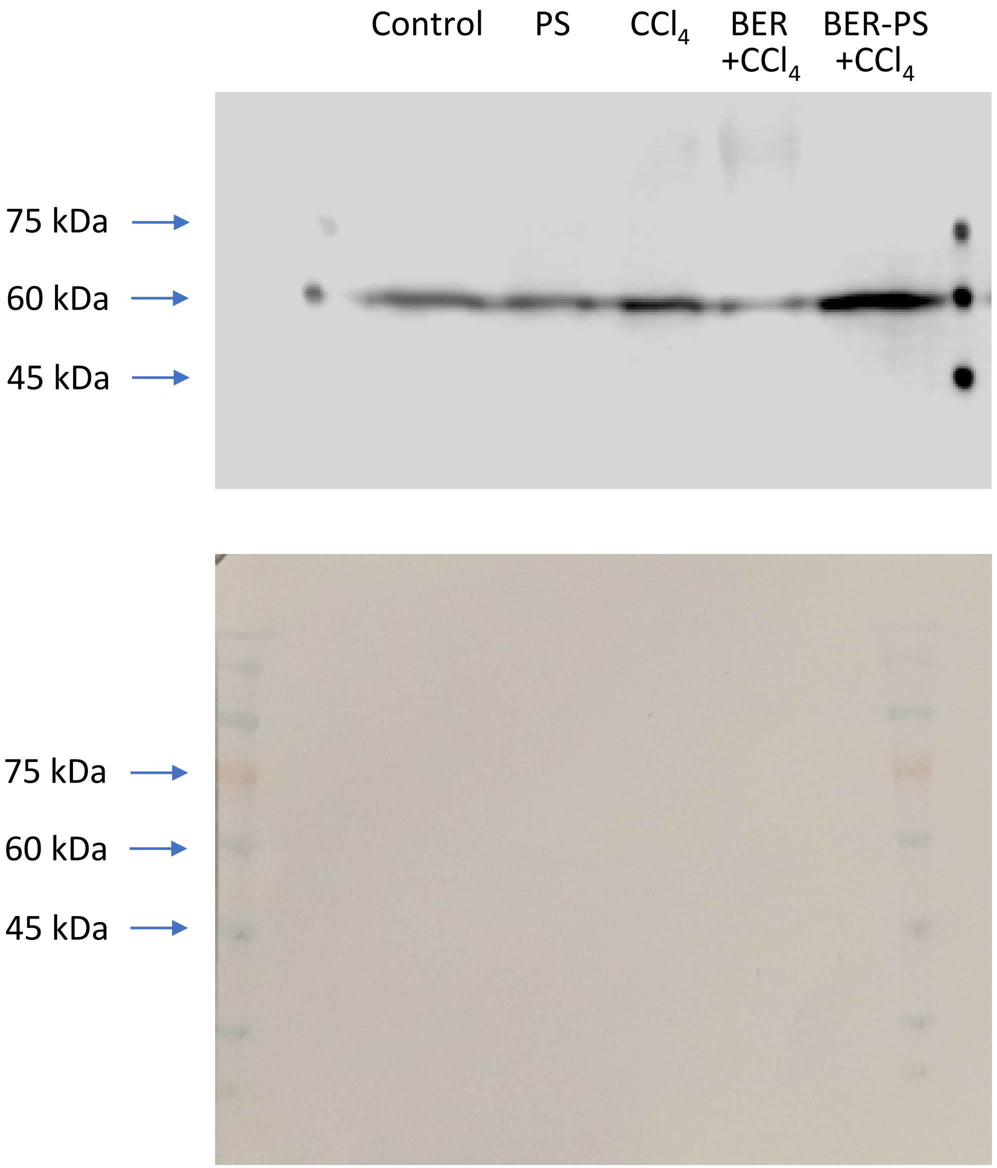
Control
PS
CCl_4
BER $+CCl_4$
BER-PS $+CCl_4$
75 kDa
60 kDa
45 kDa
75 kDa
60 kDa
45 kDa

Akt

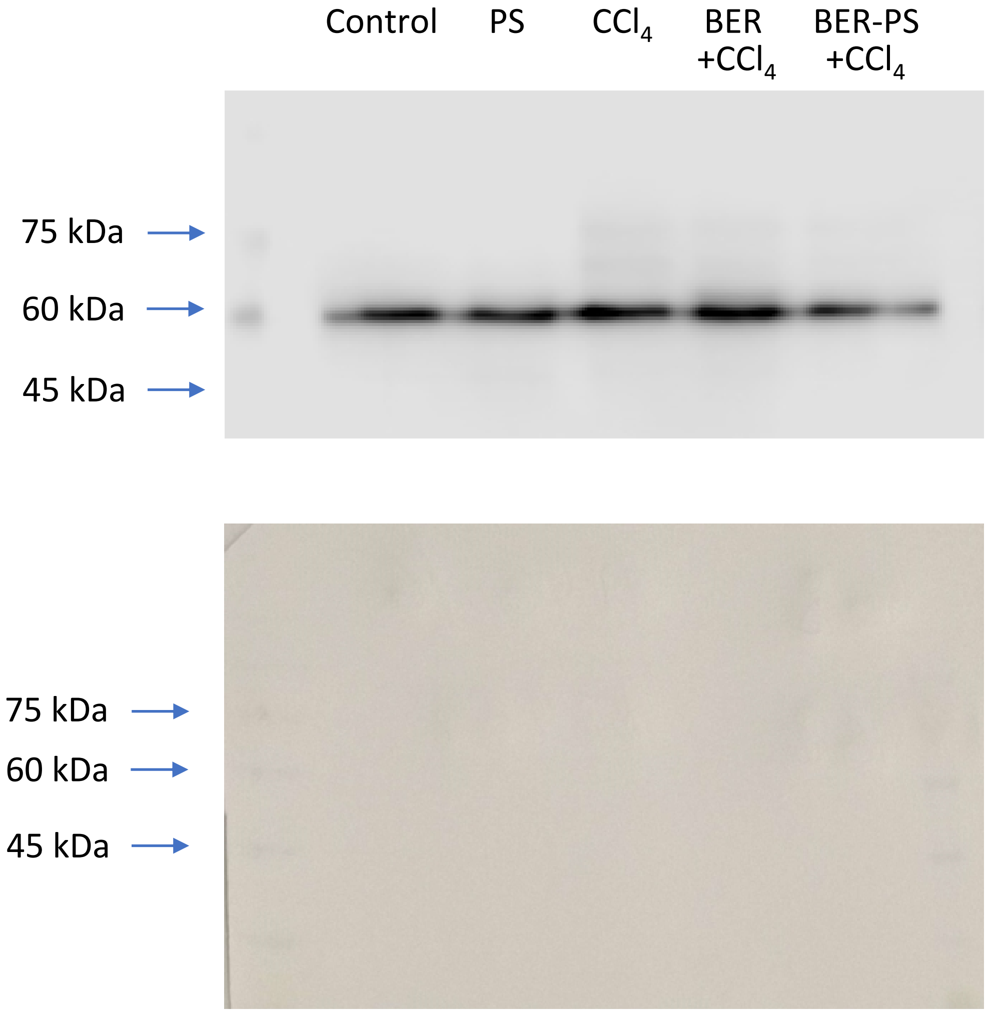

Control
PS
CCl4
BER
+CCl4
BER-PS
+CCl4
75 kDa
60 kDa
45 kDa
75 kDa
60 kDa
45 kDa

JNK + GAPDH

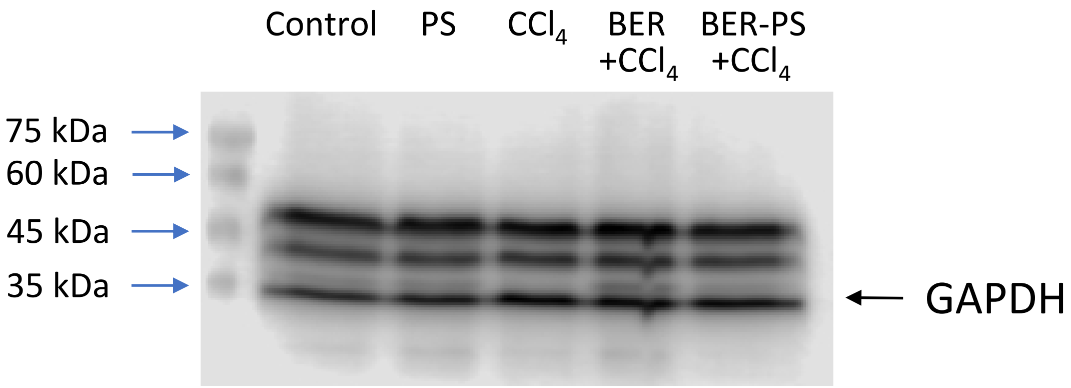
Control
PS
CCl_4
BER +CCl_4
BER-PS +CCl_4
75 kDa
60 kDa
45 kDa
35 kDa
GAPDH

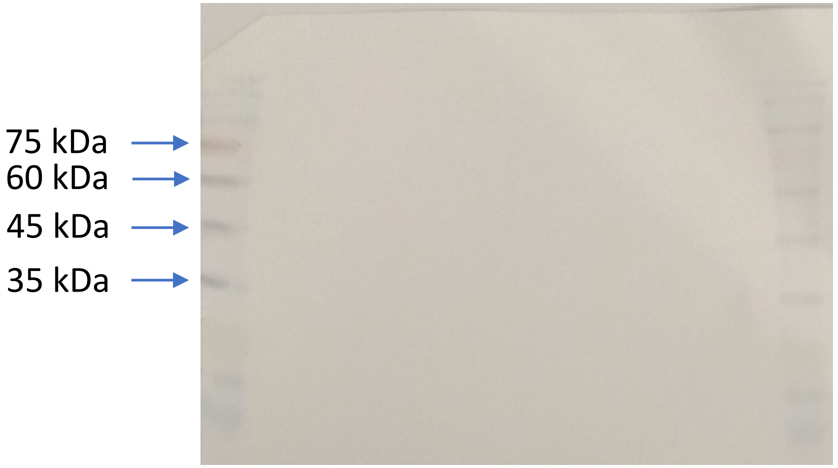
75 kDa
60 kDa
45 kDa
35 kDa
